# Vasculogenic Mimicry of HT1080 Tumour Cells *In Vivo*: Critical Role of HIF-1α-Neuropilin-1 Axis

**DOI:** 10.1371/journal.pone.0050153

**Published:** 2012-11-21

**Authors:** Roli M. Misra, Manmohan S. Bajaj, Vaijayanti P. Kale

**Affiliations:** Stem Cell Lab, National Centre for Cell Science, National Centre for Cell Science Complex, University of Pune Campus, Ganeshkhind, Pune, Maharashtra, India; University Hospital of Modena and Reggio Emilia, Italy

## Abstract

HT1080 - a human fibrosarcoma-derived cell line – forms aggressive angiogenic tumours in immuno-compromised mice. In spite of its extensive use as a model of tumour angiogenesis, the molecular event(s) initiating the angiogenic program in these cells are not known. Since hypoxia stimulates tumour angiogenesis, we examined the hypoxia-induced events evoked in these cells. In contrast to cells grown under normoxic conditions, hypoxia-primed (1% O_2_) HT1080 cells formed robust tubules on growth factor-reduced matrigel and formed significantly larger tumours in xenograft models in a chetomin-sensitive manner, indicating the role of HIF-1α-mediated transcription in these processes. Immuno-histochemical analyses of tumours formed by GFP-expressing HT1080 cells clearly showed that the tumour cells themselves expressed various angiogenic markers including Neuropilin-1 (NRP-1) and formed functional vessels containing red blood cells, thereby unambiguously demonstrating the vasculogenic mimicry of HT1080 cells *in vivo*. Experiments performed with the HT1080 cells stably transfected with plasmid constructs expressing shNRP-1 or full-length NRP-1 clearly established that the HIF1α-mediated up-regulation of NRP-1 played a deterministic role in the process. Hypoxia-exposure resulted in an up-regulation of c-Myc and OCT3/4 and a down-regulation of KLF4 mRNAs, suggesting their involvement in the tumour formation and angiogenesis. However, silencing of NRP-1 alone, though not affecting proliferation in culture, was sufficient to abrogate the tumour formation completely; clearly establishing that the hypoxia-mediated HIF-1α-dependent up-regulation of NRP-1 is a critical molecular event involved in the vasculogenic mimicry and tumor formation by HT1080 cells *in vivo*.

## Introduction

Development of a large tumour involves multiple processes, amongst which the tumour angiogenesis plays a critical role [1]. Folkman identified this phenomenon in the early nineties and underscored its importance in the chemo-radio-resistance of the tumour cells [2]. This study opened up a vast area of research in tumour biology, leading to the development of strategies that were designed to therapeutically target the angiogenic process to curtail the tumour growth [3].

Tumour angiogenesis primarily involves at least three distinct processes: formation of new blood vessels by the host-derived endothelial cells in response to the secretion of angiogenic factors by the tumour cells – the most accepted model [4], the formation of lymphatic vessels from the pre-existing lymphatic vessels – a process termed as lymphangiogenesis [5] and the formation of vascular channels lined exclusively by the tumour cells mimicking endothelial cells – a process called as vasculogenic mimicry (VM) [6]. Since the molecular mechanisms involved in the development of angiogenesis vary with the tumour type, elucidation of the process employed by a particular type of tumour forms the first line of investigation followed by the determination of the molecular mechanisms involved in the process. This is a crucial aspect in the development of anti-angiogenic strategies, as the de-differentiating tumour cells undergoing VM may not necessarily acquire sensitivity to angiogenesis inhibitors [7]–[9].

As the tumours grow in size, their microenvironment can become increasingly hypoxic. Tumours as small as 1–2 mm in diameter may show signs of hypoxia and may depend on angiogenesis for further growth [10]. Although hypoxia is toxic to normal cells, the tumour cells undergo adaptive changes that allow them to survive and even proliferate in a hypoxic environment. Over the past decade, data generated by many groups have indicated that hypoxic microenvironments contribute to the cancer progression by activating adaptive transcriptional programs that promote cell survival, motility, and tumour angiogenesis [11], [12]. Under hypoxic conditions, a signalling pathway involving a key oxygen response regulator, termed HIF (Hypoxia Inducible Factor), is switched on. HIF, a hetero-dimeric transcription factor consisting of an oxygen-sensitive α subunit (HIF-1α) and a constitutive beta subunit (HIF-1β), facilitates both oxygen-delivery and adaptation to the oxygen-deprivation by regulating the expression of a myriad of genes that control various physiological processes such as glucose uptake, metabolism, angiogenesis, erythropoiesis, cell proliferation and apoptosis [13]. HIF-1α induces the transcription of genes like PECAM, VE-Cadherin, VEGF and other proteins that stimulate the formation of blood vessels supplying nutrients to the tumour cells [12]. Vascular Endothelial Growth Factor (VEGF) is a cytokine released from the cells that promotes the neo-vessel formation and morphogenesis. The VEGF protein family consists of a number of secreted glycoproteins of which, VEGF-A, is the most widely studied member and has been implicated in both vasculogenesis and angiogenesis [14]. It acts as a mitogen as well as a chemo-attractant for the endothelial cells, thereby acting as an angiogenesis-promoting factor *in vivo* [15]. Many aggressive tumours have been shown to express gene expression signatures characteristic of human embryonic stem cells [16], [17]. Hypoxia, via HIF-1α-mediated transcription, has been shown to induce an embryonic stem cell-like transcriptional program including pluri-potency-inducing markers like OCT3/4, Nanog, c-Myc, KLF4 and SOX-2 in several cancer cell lines derived from various tissues [18]. c-Myc- deficient embryonic stem cells failed to form tumours in immune-compromised mice suggesting its essential role in tumour angiogenesis and progression [19], [20].

Another VEGF receptor, NRP-1 (Neuropilin 1), identified in the early nineties, was found to be expressed by the endothelial cells, vascular smooth muscle cells [21] as well as by the tumour cells [22]. NRP-1 is a co-receptor of VEGFR-2 and it specifically increases the binding of its ligand VEGF165, the predominant splice variant of VEGF-A playing an important role in tumour growth and angiogenesis [23], [24]. NRP-1 interacts with VEGFR-2, and increases the VEGF_165_-mediated chemotaxis of the endothelial cells [25]. Growing evidence supports a critical role of these receptors in the tumour progression. NRP-1 expression is found to be up-regulated in multiple tumour types and correlates with tumour progression and/or prognosis in a tumour-specific manner [26]. NRP-1 may either mediate its effects on the tumour progression indirectly by promoting tumour angiogenesis or directly by affecting the biology of the tumour cells themselves [27]. VEGF produced by tumor cells has been shown to act in an autocrine manner to promote cell growth through interaction with NRP-1 [28].

Human sarcomas often show areas of central necrosis and hypoxia [29]. Detwiller *et al* examined oligonucleotide micro-arrays from 38 human sarcoma tumours and 14 normal tissues and found that sarcomas have a distinctly different pattern of hypoxia-related gene expression with an up-regulation of several genes including HIF-1α and VEGF [30]. Since VEGF is considered as a critical factor for tumour angiogenesis, an attempt has been made to silence the VEGF by siRNA-mediated approach to control the tumour growth in an experimental model system [30]. A complete abrogation of VEGF secretion slowed down the tumour growth but did not prevent the tumour formation by the human fibrosarcoma-derived HT1080 cells, indicating the involvement of other mechanisms in the tumour growth.

Although the HT1080 cells are used as a model system in several studies, the crucial molecular events involved in their angiogenic behaviour have not been identified. In the present study, we used hypoxia-primed HT1080 cells as a model system to elucidate the molecular mechanisms involved in the aggressive angiogenic tumour formation by them.

## Results

### HT1080 Tumours are Highly Angiogenic and Show *in situ* Hypoxia

**Figure 1:**
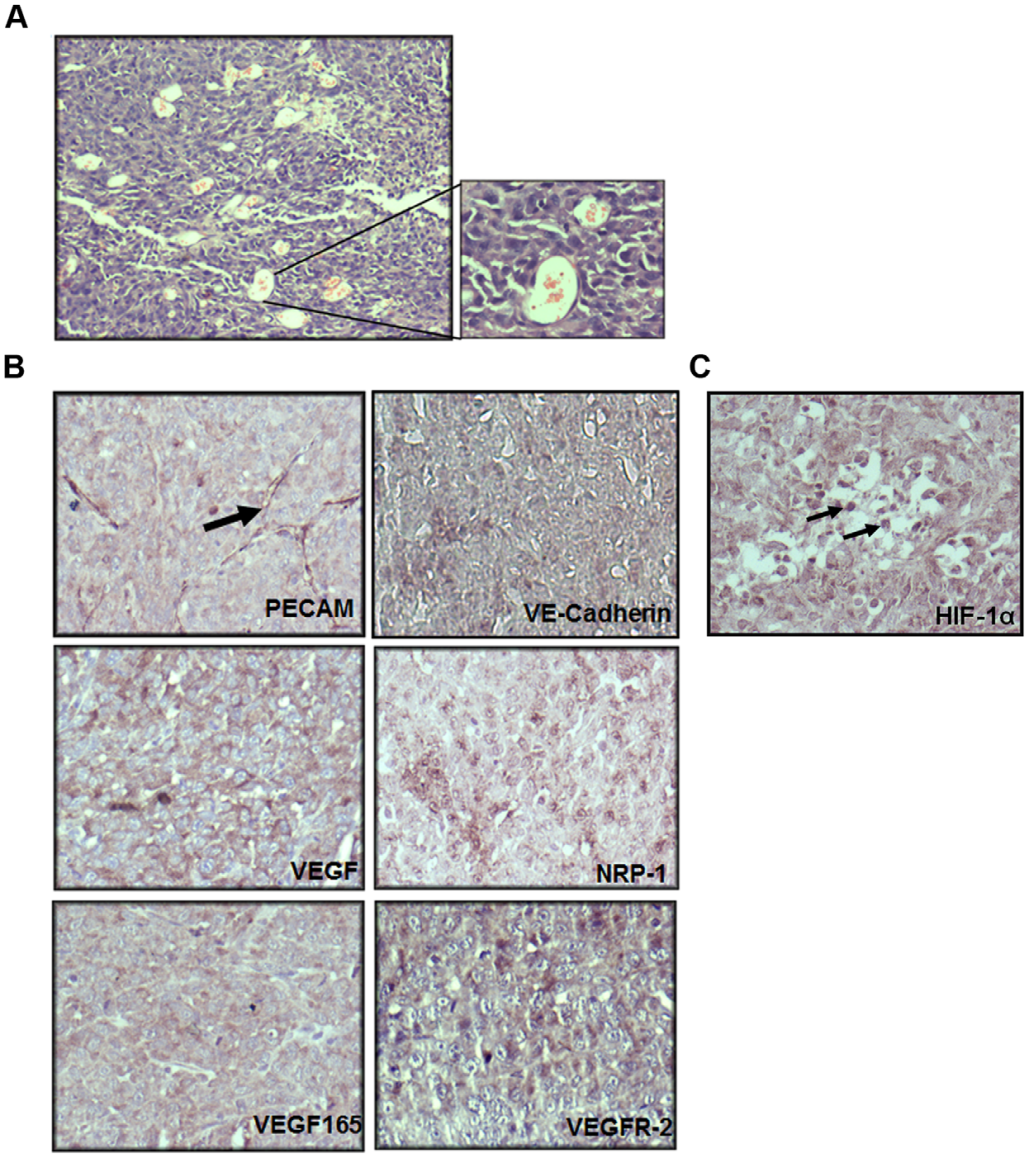
HT1080 tumours are highly angiogenic and show presence of *in situ* hypoxia. A. An image of a haematoxylin and eosin (HE)-stained section of HT1080 tumours showing the presence of abundant vascular channels containing red blood cells is illustrated. A magnified image of these vascular channels is depicted on the right hand side. (Original magnifications 100X and 400X respectively) **B.** Paraffin sections of HT1080 tumours were subjected to immuno histochemistry (IHC) analyses using antibodies to various angiogenic markers. The tumour cells were positive for PECAM, VE-Cadherin, VEGF, NRP-1, VEGF_165_ and VEGFR-2 (violet colour). PECAM positive micro-vessel formation is indicated by a black arrow (top layer, right panel). **C.** Nuclear localization of HIF-1α in the tumour cells confirms the presence of *in situ* hypoxia. The sections were counterstained with hematoxylin to demarcate the nuclei (blue).

The HT1080 cells formed aggressive tumours when injected subcutaneously in the flanks of the immuno-compromised mice. The Hematoxylin-Eosin (HE)-stained sections of the HT1080 tumours showed the presence of several vascular channels harbouring red blood cells (RBCs) that are indicative of high levels of angiogenesis (Figure. 1A). Immuno-histochemical (IHC) analyses of these sections revealed that the tumour cells themselves expressed various angiogenic markers like PECAM, VE-Cadherin, VEGF, VEGF_165_, NRP-1 and VEGFR-2 (FLK1/KDR) (Figure. 1B), suggesting that the HT1080 cells had acquired a vascular-like phenotype *in vivo*. Nuclear stabilization of HIF-1α in the tumor cells confirmed the presence of the *in situ* hypoxia (Figure. 1C).

### Hypoxia Stimulates Proliferation of HT1080 Cells

**Figure 2:**
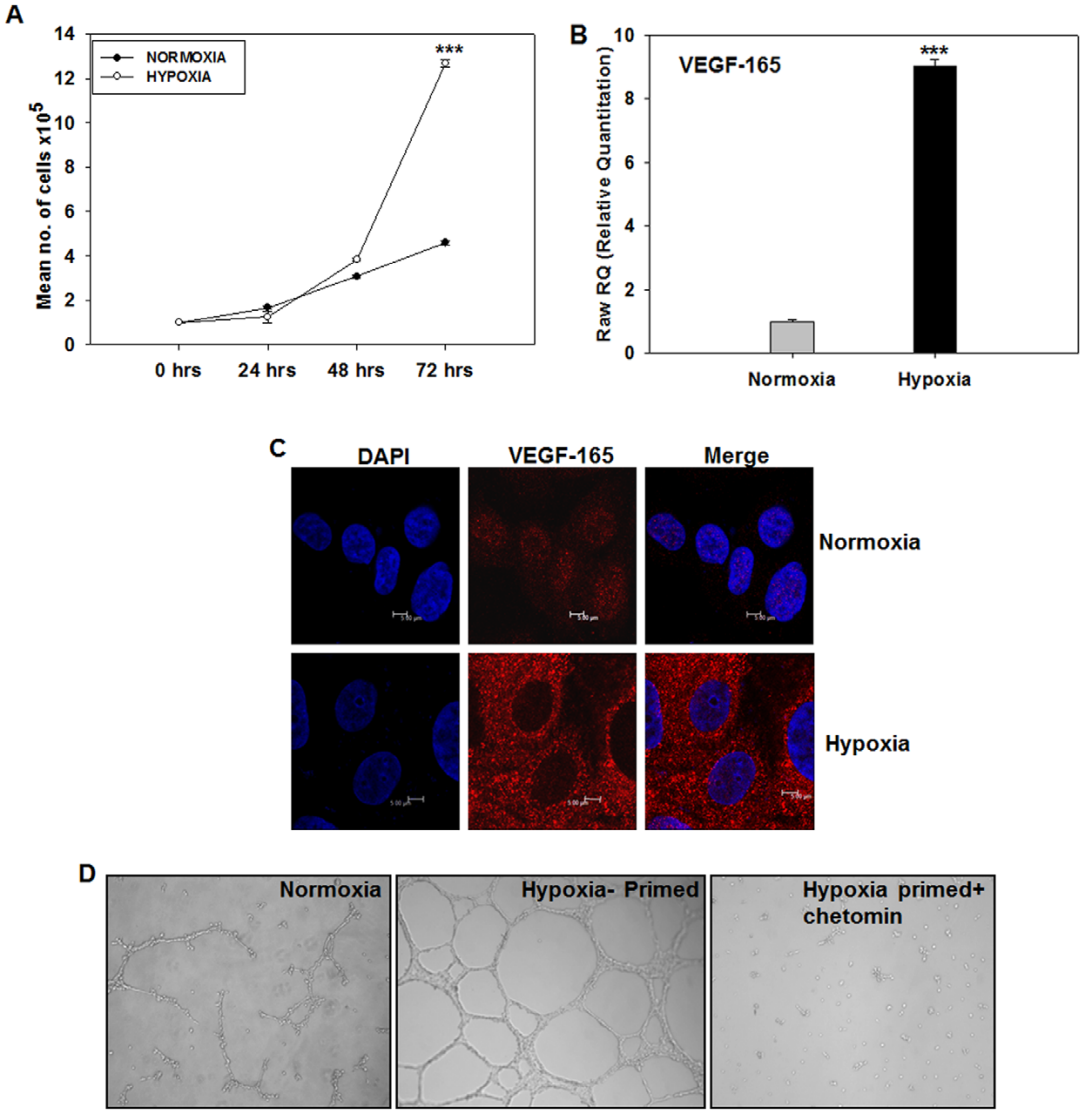
HT1080 cells respond to hypoxia by up-regulation of growth-promoting cytokine, VEGF_165_. A. Growth kinetics of the HT1080 cells was studied under normoxia and hypoxia. Cell proliferation rate was higher under hypoxic conditions as compared to the normoxic conditions. **B.** Quantitative PCR experiments were performed on the HT1080 cells incubated under normoxia and hypoxia for quantification of VEGF_165_ mRNA. The hypoxic cells showed ∼9 folds higher expression of VEGF_165_ mRNA compared to the normoxic ones (6 hours time point, N = 3, *** P<0.001). **C.** Confocal microscopy analysis of the cells incubated under normoxia vs. Hypoxia (48 hours) using an antibody to VEGF_165_ shows that the hypoxic cells secret this growth-promoting cytokine at high levels. Nuclei are demarcated by DAPI (Blue) **D.** Consistent with the high level of VEGF_165_ at gene and protein levels, the hypoxia-primed HT1080 cells undergo a robust tubule formation on growth factor-reduced matrigel (middle panel) compared to the normoxic cells (left hand panel). The tubule formation was sensitive to the presence of chetomin in the medium (right hand panel) indicating that the HIF1-α-mediated transcription is involved in the process. (Original magnification: 40 X).

In order to elucidate the mechanistic aspects of the hypoxia-mediated activation of angiogenic program using HT1080 cells as a model system, it was necessary to ascertain whether these cells could survive and grow under hypoxia. As seen in the Figure. 2A, the HT1080 cells incubated in a hypoxia chamber (1% oxygen; hypoxic) showed an enhanced growth rate compared to the cells incubated under normoxia (normoxic). The enhanced proliferation became evident by 48 hours and peaked at 72 hours, indicating that the phenotypic response to hypoxia was clearly evident within 48 hours of hypoxic induction. Since VEGF_165_ is known to act as a growth-promoting cytokine under hypoxic conditions [31], we quantified the VEGF_165_ mRNA in these cells by performing real time PCR experiments. We observed a ∼9 fold up-regulation of VEGF_165_ mRNA in the hypoxic cells as compared to the normoxic ones (Figure. 2B; *** p<0.001;). Immunofluorescence experiments further revealed a significant up-regulation of this cytokine under hypoxia at protein level as well (Figure. 2C). Interestingly, the normoxic cells showed a nuclear localization of VEGF_165_ while the hypoxic cells exhibited abundant cytoplasmic localization. Stretches of basic amino acids that could potentially act as nuclear localization sequences have been identified in VEGF_165_ sequence [32]. It may be interesting to examine whether the cytoplasmic VEGF_165_ arises from a different usage of initiation codon in response to hypoxia as against the nuclear VEGF_165_ seen under normoxia and whether their angiogenic activity correlates with this localization pattern [33].

These data showed that the HT1080 cells cultured under hypoxia (hypoxia-primed) for 48 hours form a suitable model to study the hypoxia-mediated molecular events involved in tumour angiogenesis and growth.

### Hypoxia-primed Cells Show Enhanced Tubulogenesis on Matrigel in a HIF-1α-dependent Manner

Tubule formation on matrigel is an excellent *in vitro* correlate of angiogenesis [34]. Hypoxia-mediated activation of HIF-1α has been shown to drive tubule formation on matrigel [35], [36]. We, therefore, examined whether hypoxia-primed HT1080 cells exhibit an enhanced tubulogenesis on matrigel. It was observed that the hypoxia-primed cells started forming the tubules at a much earlier time point compared to their normoxic counterpart (3 hrs vs. 6 hrs respectively) and formed a denser network of tubes (Figure. 2D - middle panel). Quantification of the tubule length at various time points showed that the hypoxia-primed cells formed significantly longer tubules compared to the normoxic cells (Figure. S1A).

We then ascertained whether the enhanced tubule-formation by the hypoxia-primed cells was a HIF1α-dependent or -independent [37] process. Incorporation of Chetomin (100 nM), a well-known pharmacological inhibitor of HIF-1α that blocks the binding of p300 subunit with the HRE region of HIF-1α, thereby blocking the transcription of downstream genes [38], in the incubation medium resulted in a complete abrogation of tubule formation by the hypoxia-primed cells (Figure. 2D, right-hand panel, Figure. S1A), confirming the role of HIF-1α-mediated transcription in the process. In order to examine whether hypoxia also enhances the tubule formation ability of any other tumour cell line, we seeded normoxia- and hypoxia-primed MDA-MB-231 breast cancer cells on matrigel. Chetomin was added in one of the sets incubated under hypoxia. It was observed that the hypoxia-primed cells formed robust tubules on matrigel (Figure. S1B) in a chetomin-sensitive manner, suggesting that hypoxia-mediated enhancement of tubulogenesis may not be a specific property of HT1080 cells alone.

### HT1080 Cells Exhibit Vasculogenic Mimicry *in vivo*

**Figure 3:**
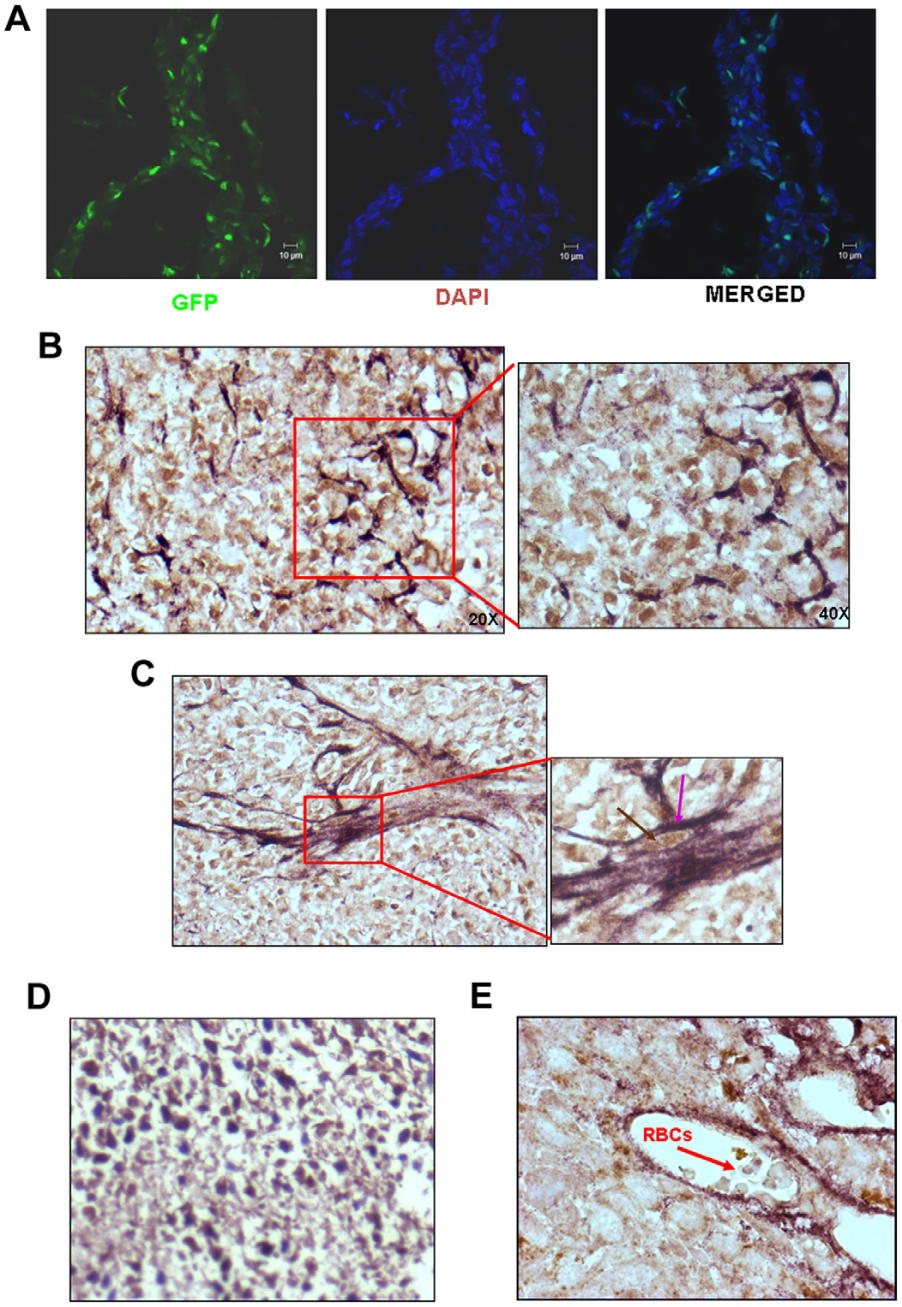
HT1080 tumour cells employ vasculogenic mimicry to accommodate *in situ* hypoxia. A. Visualization of GFP^+^ tumour cells in the cryosections of the tumours formed by HT1080/WT/GFP cells showed that the tumour cells themselves form vessels that run criss-cross in the tumour mass. DAPI was used to demarcate the nuclei (bar - 10 µm) **B.** Double IHC experiments performed on the paraffin sections of the tumours formed by HT1080-GFP cells with anti-GFP and anti-PECAM antibodies show that the entire tumour mass was filled with GFP^+^ tumour cells with the vessels formed by the double positive cells. The intensity of PECAM staining was maximal in the cells forming tubes. (Original magnification 200X). A part of the image has been magnified to show the details (original magnification 400 X). **C.** A vessel formed by the double positive cells is shown (original magnification 200X). The inset clearly shows that the cells forming the vessels have brown nuclei (GFP – indicated by a brown arrow) and violet border (PECAM – indicated by a violet arrow) (original magnification 630 X). **D.** Image shows absence of PECAM staining in the tumour section of HT1080 where anti-PECAM antibody has not been applied; whereas the GFP signal is clearly seen (Original magnification is 200X). **E.** A cross section of the blood vessel formed by GFP-PECAM double positive cells is depicted. Presence of red blood cells in the lumen is marked by a red arrow (original magnification 630X).

Many types of cells form tubules on matrigel. It is, however, necessary to show that they themselves form functional blood vessels *in vivo*. In order to clearly distinguish between the tumour cells and the host-derived endothelial cells, we subcutaneously injected HT1080 cells expressing a reporter gene, GFP (Figure. S1C) in NOD/SCID mice and both cryosections and paraffin sections were prepared from them. A visual inspection of a cryosection under confocal microscope clearly showed that the GFP^+^ tumour cells formed vessel-like structures running criss-cross in the tumor mass (Figure. 3A). We then performed a double IHC experiment with the paraffin sections using antibodies to GFP and PECAM. As seen in the Figure. 3B, the entire tumour mass was formed by GFP^+^ cells (brown nuclei). The GFP^+^ cells themselves expressed PECAM on their surface (violet borders, Figure. 3C, inset and Figure. 3D); unambiguously showing that the HT1080 cells themselves had acquired a vascular cell-like phenotype. It was important to note that the PECAM staining was very prominent in the GFP^+^ cells that participated in the formation of the tube-like structures. The tube-like structures formed by the double positive cells were seen to harbour red blood cells indicating that they represented blood-conducting vessels (Figure. 3E). The data confirmed that the HT1080 cells exhibited vasculogenic mimicry *in vivo*.

### Hypoxia Up-regulates Angiogenic Program in the HT1080 Cells

**Figure 4:**
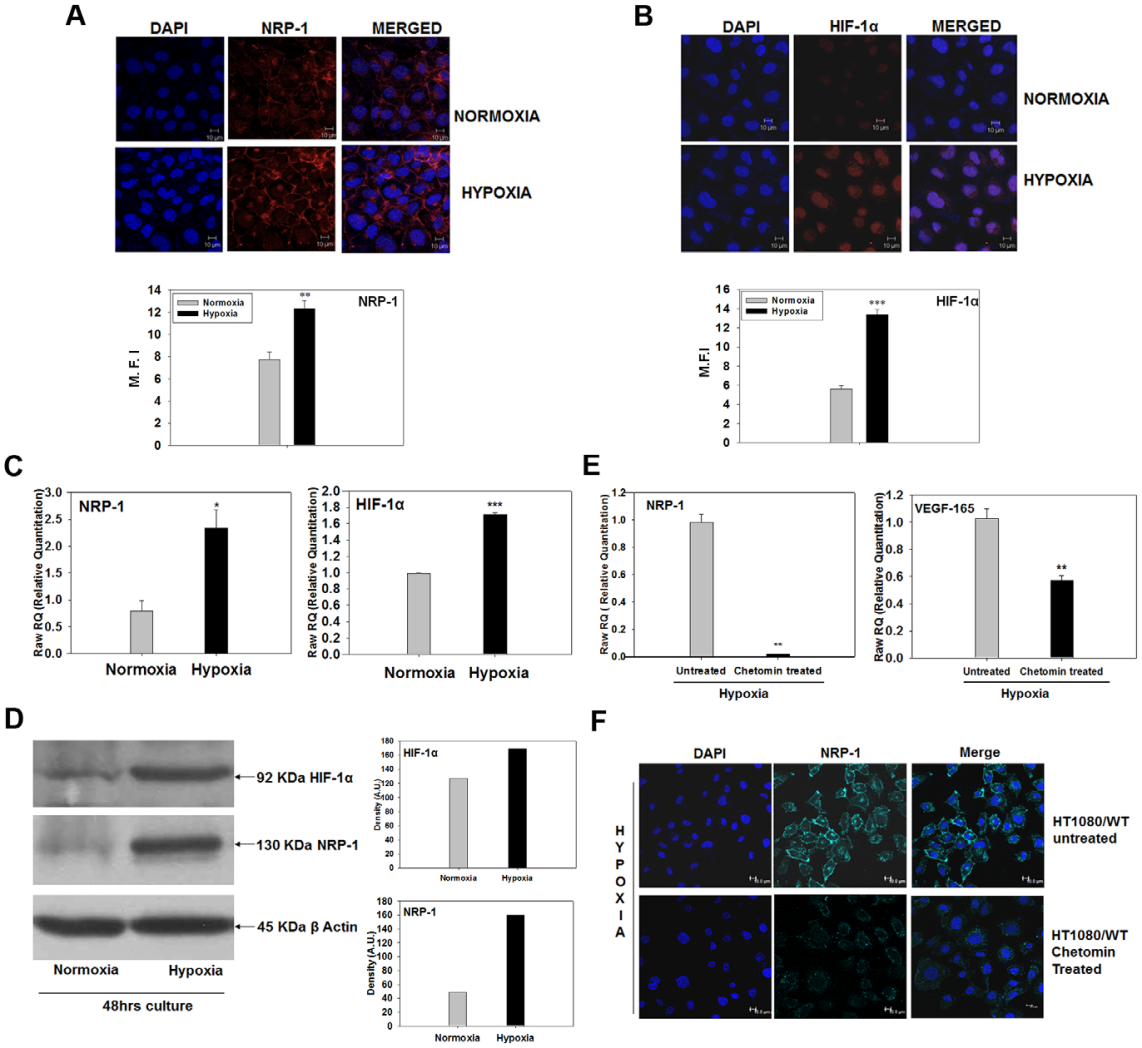
Hypoxia up-regulates angiogenic program in HT1080 cells. Confocal microscopy analyses show that the hypoxic cells exhibit up-regulation of NRP-1 (**A**) and nuclear stabilization of HIF-1α (**B**). Nuclei are demarcated by DAPI (Blue). Mean fluorescence intensity (M.F.I.) of the cells was measured by Image J software (NIH) at membrane (for NRP-1) and in the nuclear region (for HIF-1α). The M.F.I. of 30 randomly selected cells was used to calculate mean ± S.E.M. The analyses have been graphically depicted (b and d for NRP-1 and HIF-1α respectively) ** p<0.01 and *** p<0.001. **C.** Quantitative PCR analyses for NRP-1 and HIF-1α mRNA show 2.4 and 1.7 folds up-regulation of these genes in the cells incubated under hypoxia compared to normoxia. (N = 3; *p<.05 and ** p<.01). **D.** Western blot experiments performed on the cells grown under normoxia vs. hypoxia show that the protein levels of both HIF-1α (upper panel) and NRP-1 (middle panel) are up-regulated in the hypoxic cells compared to the normoxic ones (∼1.4 and 3.2 folds respectively). **E.** Results of quantitative PCR experiments showed that the hypoxia-induced up-regulation of NRP-1 and VEFG_165_ mRNA was sensitive to the presence of chetomin in the medium, indicating that these genes are down-stream events in the HIF-1α-mediated transcription process. **F.** Confocal microscopy analysis shows that the hypoxia-mediated up-regulation of NRP-1 protein (Cyan, upper panel) is abrogated in the presence of chetomin in the medium (lower panel), suggesting that NRP-1 expression critically depends on the HIF-1α -mediated transcription. Nuclei are demarcated by DAPI (Blue).

NRP-1 has been identified as a co-receptor for VEGF_165_ and is known to play an important role in the angiogenic process [25], [39]. The enhanced expression of VEGF_165_ by the hypoxia-primed HT1080 cells, the HIF-1α-dependent tubule formation seen in the earlier experiments and the formation of vessel-like structures by the tumour cells *in vivo* prompted us to look for the expression of NRP-1 in the hypoxia-primed cells. We performed immunofluorescence staining experiments on both hypoxic and normoxic cells using an antibody to NRP-1. The hypoxic cells showed a clear up-regulation of NRP-1 at the cell membrane (Figure. 4A ** p<0.01). As expected, the nuclear stabilization of HIF-1α was also seen in the hypoxic cells (Figure. 4B, ***p<0.001).

Real time PCR experiments performed to quantify the NRP-1 mRNA showed that the expression of NRP-1 was 2.4 folds higher in the hypoxic cells compared to the normoxic cells (Figure. 4C left hand panel, *p<0.05). The level of HIF-1α mRNA was also found to be up-regulated (1.7 folds high) by the hypoxia (Figure. 4C right hand panel, ***p<0.001). Data obtained in the Western blot experiments performed on these cells revealed that the expression of both these molecules was highly up-regulated under hypoxia at translational level as well (Figure. 4D, ∼1.4 folds for HIF-1α and 3.2 folds for NRP-1).

The up-regulation of NRP-1 and VEGF_165_ mRNA by hypoxia was found to be sensitive to the presence of chetomin in the medium (Figure. 4E), confirming that in the HT1080 cells hypoxia-induced up-regulation of NRP-1 and its ligand, VEGF_165,_ was dependent on the HIF-1α- mediated transcription. Similar results were also obtained in immunofluorescence experiments, thereby confirming the real time PCR data (Figure. 4F). We then examined whether hypoxia also induces the tubulogenic property on non-neoplastic cells by using MC3T3#24 cells. The hypoxia-primed MC3T3#24 cells neither showed any tubule formation on matrigel nor did their NRP-1 expression change after hypoxia-exposure (data not shown), suggesting that the effects of hypoxia were perhaps tumour cell-specific.

These data showed that hypoxia up-regulates the expression of two important angiogenic markers, NRP-1 and VEGF_165_, in the HT1080 cells in a HIF-1α-dependent manner.

NRP-2 is also known to bind to VEGF and participate in tumor angiogenesis and growth [40]. Therefore, we quantified the expression of NRP-2 mRNA in the HT1080 cells incubated under both normoxic and hypoxic conditions. We observed that the level of NRP-2 was not affected by hypoxia (Figure. S1D), suggesting that perhaps it did not have a role in the hypoxia-mediated angiogenic behavior of these cells.

### Generation and Characterization of HT1080 Clones Stably Expressing shNRP-1 and fl/NRP-1

**Figure 5:**
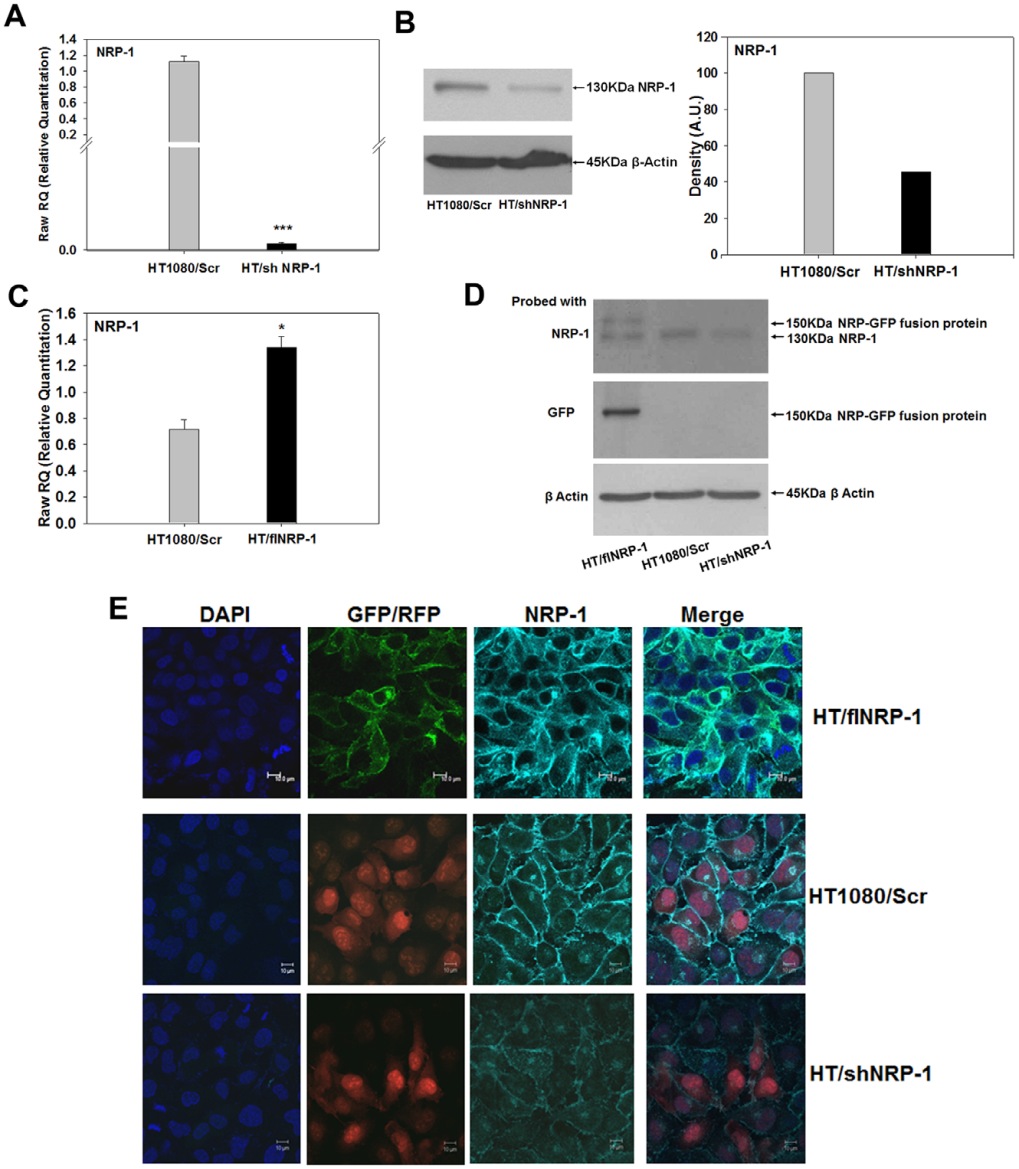
Characterization of HT/shNRP-1 and HT/flNRP-1 clones. A. Silencing of NRP-1 expression in the HT/shNRP-1 cells was validated at transcript level by performing quantitative PCR experiments. The NRP-1 mRNA was ∼500 folds down-regulated in the shNRP-1 clone (N = 3; ***p<0.001) showing that the shRNA constructs used were effective. **B.** The results obtained in the PCR experiments were validated by western blot experiments. The NRP-1 expression was down-regulated in the HT/shNRP-1 cells at protein level as well. **C.** Quantitative PCR analyses confirmed the higher expression of NRP-1 transcript in the HT/flNRP-1 clone compared to the HT1080/Scr cells (∼2 folds, N = 3; *p<0.05). **D.** Western blot experiments were performed on the HT1080/Scr, HT/shNRP-1 and HT/flNRP-1 cells. Data show the presence of a 150 kDa band of the NRP-GFP fusion protein in the HT/flNRP-1 cells when the blot was probed with antibodies to NRP-1 (upper panel) and GFP (middle panel). The down-regulation of NRP-1 in the HT/shNRP-1 clone is also seen (upper panel). **E.** Confocal microscopy analyses show a higher expression of NRP-1 in the HT/flNRP-1 clone (upper panel) and a reduced expression of NRP-1 in HT/shNRP-1 clone (Lower panel) compared to the HT1080/Scr cells (middle panel). Membrane-localized NRP-1-GFP fusion protein is seen in the HT/flNRP-1 cells. The RFP fluorescence is seen in the nuclei of HT1080/Scr and HT/shNRP-1 cells respectively. Nuclei are demarcated by DAPI (Blue).

In order to validate the role of NRP-1 in the hypoxia-induced angiogenic behavior of the HT1080 cells, we developed stable clones of these cells expressing NRP-1-specific shRNAs (HT/shNRP-1) on one hand and a full-length NRP-1 (HT/flNRP-1) on the other. The cells transfected with the vector expressing scrambled sequences were used as control (HT1080/Scr). There was no difference in the growth kinetics of these cells as compared to the wild type or the HT1080/Scr cells (data not shown) indicating that NRP-1 does not play any role in the *in vitro* growth properties of the HT1080 cells. These clones were characterized by performing real time PCR, western blot and immunofluorescence experiments to ascertain the NRP-1 expression levels before their use in further experiments (Figure. 5A–E).

### NRP-1 Controls the Hypoxia-induced Angiogenic Properties of HT1080 Cells

**Figure 6:**
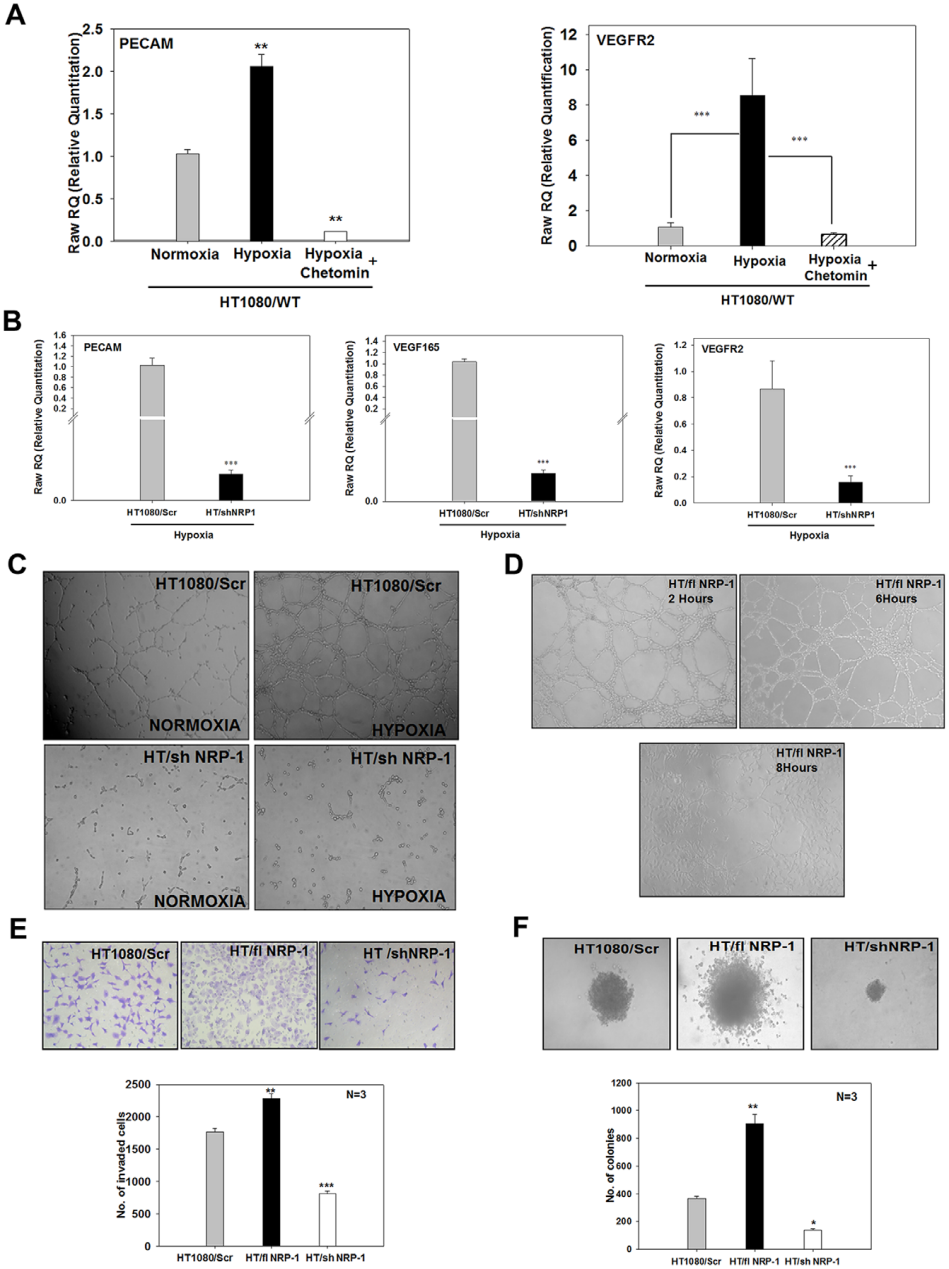
NRP-1 controls the hypoxia-induced angiogenic program in HT1080 cells. A. Expression of PECAM and VEGFR-2 mRNA was quantified by performing real time PCR experiments on the HT1080/WT cells that were incubated under normoxia and hypoxia – with or without chetomin. Both PECAM and VEGFR-2 mRNAs were up-regulated by hypoxia. Presence of chetomin abrogated the up-regulation of PECAM and VEGFR2 genes by hypoxia indicating that these genes are regulated by the HIF-1α-mediated transcription. **B.** Quantitative PCR experiments performed on the HT1080/Scr and HT/shNRP-1 cells incubated under hypoxia show that hypoxia failed to up-regulate the expression of PECAM, VEGF_165_ as well as VEGFR-2 in the HT/shNRP-1 cells, indicating that the hypoxia-induced angiogenic program in the HT1080 cells is controlled by NRP-1. **C.** Tubule formation on matrigel by HT1080/Scr and HT/shNRP-1 cells under normoxia and hypoxia is depicted. The HT/shNRP-1 cells failed to form tubules even after priming with hypoxia (lower right hand panel), indicating that NRP-1 expression in critical for the enhanced tubule formation by the hypoxia-primed HT1080 cells. The hypoxia-primed wild type cells formed robust tubules as seen in the earlier experiments (upper right hand panel). **D.** HT/flNRP-1 cells form dense tubules even without the hypoxia-priming. The tubule formation by these cells started very early (2 hours) and became very dense by 6 hours (upper right hand panel). After 6 hours, the tubules collapsed as the HT/flNRP-1 cells invaded the matrigel vigorously (lower panel) forming a monolayer in the well. **NRP-1 controls the tumorigenic properties of HT1080 cells. E.** Matrigel-invasion property of HT1080/Scr cells was compared with that of HT/flNRP-1 and HT/shNRP-1 cells. A representative image of the invaded cells stained with crystal violet is depicted (Original magnification: 100X). Quantification of the invaded cells showed that the HT/flNRP-1 cells possessed significantly enhanced invasive property (N = 3; ** p<0.01) while the HT/shNRP-1 cells showed a significantly reduced invasive ability (N = 3; ***p<0.001). **F.** Anchorage-independent growth of HT1080/Scr, HT/shNRP-1 and HT/flNRP-1 was examined by performing soft agar colony assay. A representative phase contrast image of the colonies formed by these cells is illustrated (original magnification: 100X). The HT/flNRP-1 cells formed large colonies having loose migrating cells at the border (middle panel), while the HT/shNRP-1 cells formed very small compact colonies. Quantification of the colony formation shows that the number of colonies formed by the HT/flNRP-1 cells was significantly higher (**p<0.01) while that by the HT/shNRP-1 cells was significantly lower (* p<0.05) compared to the HT1080/Scr cells. Data show that NRP-1 expression is necessary for the anchorage-independent growth of HT1080 cells. Data are represented as mean ± S.E.M.

PECAM, VEGF_165_ and VEGFR-2 are some of the important signature molecules involved in the tumour angiogenesis. Quantitative PCR experiments performed on the normoxia- or hypoxia-primed HT1080/WT cells – with or without chetomin – showed that up-regulation of PECAM and VEGFR-2 mRNA in response to hypoxia was dependent on HIF1α-mediated transcription (Figure. 6A). Since in our earlier experiments we had observed that hypoxia-mediated up-regulation of NRP-1 was also chetomin-sensitive, we examined whether hypoxia regulates the expression of these angiogenic markers through NRP-1. Quantitative PCR analyses of PECAM, VEGF_165_ and VEGFR2 mRNA (Figure. 6B) in HT1080/Scr and HT/shNRP-1 cells grown under hypoxia revealed that hypoxia failed to up-regulate the expression of these genes in the HT/shNRP-1 cells. These data clearly showed that the expression of these angiogenic markers was a down-stream event of the HIF-1α-NRP-1 axis in the HT1080 cells.

It became imperative to determine whether silencing of NRP-1 also affects the hypoxia-mediated enhancement of tubule formation by the HT1080 cells. The hypoxia−/normoxia-primed HT1080/Scr and HT/shNRP-1 cells were seeded on polymerised growth factor-reduced matrigel. It was observed that consistent with their lack of angiogenic gene expression seen in the earlier experiments, the HT/shNRP-1 cells failed to undergo tubulogenesis even after hypoxia-priming (Figure. 6C-lower panel), whereas the hypoxia-primed HT1080/Scr cells showed a higher degree of tubule formation (Figure. 6C –upper panel). The average length of the tubules formed by the hypoxia-primed HT1080/Scr cells was significantly longer than those formed by the normoxic ones (Figure. S1E). The HT/flNRP-1 cells on the other hand formed a dense network of tubules very early (<2 hrs) even without the hypoxia-priming (Figure. 6D, upper panels). The length of the tubules formed by HT/flNRP-1 cells at 4 hours was more than the length of those formed by the HT1080/Scr cells at 6 hours (Figure. S1F). This network remained stable for 6hours and then collapsed as these cells invaded the matrigel very aggressively and formed a monolayer on the surface of the wells (Figure. 6D-lower panel).

These data clearly show that NRP-1 acts as a pivotal determinant of the *in vitro* angiogenic properties of the HT1080 cells.

### NRP-1 Participates in the *in vitro* Tumorigenic Properties of HT1080 Cells

The invasive behaviour of HT/flNRP-1 cells on matrigel showed that an exogenous expression of NRP-1 confers significantly enhanced matrigel-invasion ability on the HT1080 cells. We, therefore, examined whether silencing of NRP-1 compromises this property. As seen in the Figure. 6E (right panel and bar diagram), the HT/shNRP-1 cells showed a significantly reduced ability to invade the matrigel compared to the HT1080/Scr cells (left panel and bar diagram; *** p<0.001). Consistent with the earlier observation made in the tubulogenesis experiment, the HT/flNRP-1 cells invaded the matrigel in significantly larger numbers (Figure. 6E -middle panel and bar diagram, ** p<0.01) compared to the HT1080/Scr cells (left panel and bar diagram).

Anchorage-independent growth is yet another *in vitro* correlate of tumorigenesis. We seeded HT1080/Scr, HT/shNRP-1 and HT/flNRP-1 cells in soft agar (0.3%), and evaluated the colony formation after 4 weeks. As seen in the Figure. 6F, the HT/shNRP-1 cells formed very small and fewer colonies in the soft agar (right panel and bar diagram) compared to the HT1080/Scr cells (left panel and bar diagram) indicating that their anchorage-independent growth capacity was severely compromised. Quantification of the colony formation showed that the result was statistically significant (Figure. 6F lower panel; **p<0.01). The HT/flNRP-1 cells formed very large and numerous colonies having loose borders (Figure. 6F, middle panel and bar diagram; **p<0.01; Figure. S2A) compared to the HT1080/Scr and HT/shNRP-1 cells (Figure. 6F left panel, right panel and bar diagram.). About 8–9% of the seeded cells (1×10^4^) formed colonies in HT/flNRP-1 set as against ∼3–4% in HT1080/Scr and ∼ 1% in HT/shNRP-1 sets (Figure. S2B) showing that an exogenous expression of NRP-1 increased the clonogenic capacity of HT1080 cells. These data clearly show that in addition to mediating the HIF-1α-mediated angiogenic program, NRP-1 also enhanced the anchorage-independent growth properties and matrigel-invasion capacity of the HT1080 cells.

### NRP-1 Plays a Crucial Role in Tumour Growth and Angiogenesis of HT1080 Cells *in vivo*

**Figure 7:**
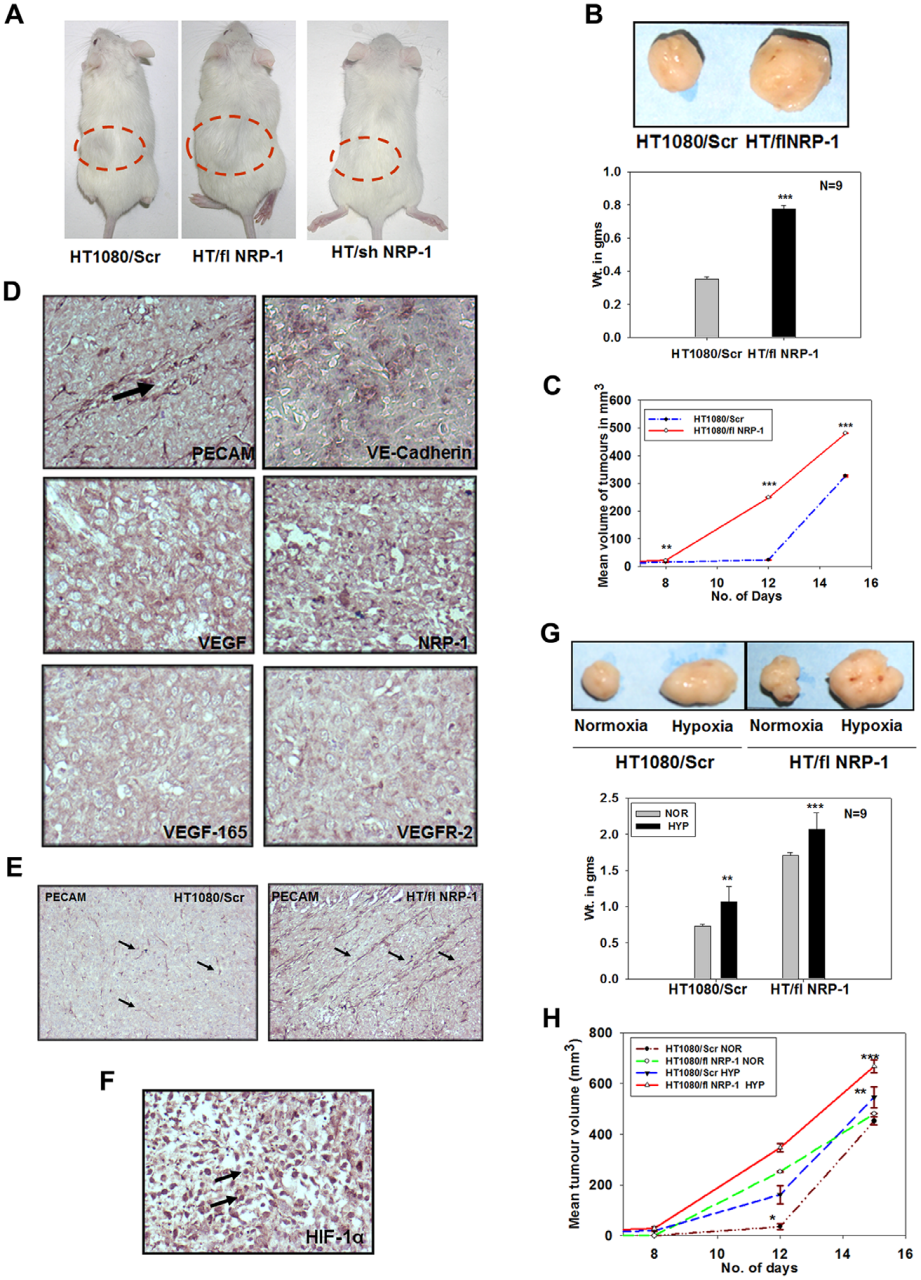
NRP-1 plays a crucial role in tumour growth and angiogenesis *in vivo.* A. HT1080/Scr, HT/flNRP-1 and HT/shNRP-1 cells were injected subcutaneously in the flanks of NOD/SCID mice (9 mice per set) to compare their tumorgenic potential. HT/flNRP-1 cells formed larger tumours (mouse in the middle) compared to those formed by the HT1080/Scr cells (mouse on the left). HT/shNRP-1 cells did not form tumour (mouse on right, 0/9). **B.** A comparative image of the tumours formed by the HT1080/Scr and the HT/flNRP-1 cells is depicted. The tumours formed by HT1080/Scr and HT/flNRP-1 cells were weighed. The mean weight of the tumours formed by the HT/flNRP-1 was significantly higher than that of the HT1080/Scr cells. (N = 9, ***p<0.001) **C.** The kinetics of tumour formation by the HT/flNRP-1 cells was significantly higher compared to that of HT1080/Scr cells (**p<0.01, ***p<0.001). **D.** HT/flNRP-1 tumor sections were subjected to IHC analyses using antibodies to various angiogenic markers like PECAM, VE-Cadherin, VEGF, VEGF_165_, NRP-1 and VEGFR-2. The sections showed a strong expression of all these markers indicating that the HT/flNRP-1 tumours are highly angiogenic (original magnification: 200X). **E.** Paraffin sections of the tumours formed by the HT1080/Scr and the HT/flNRP-1 were immuno-stained with an anti-PECAM antibody. The HT/flNRP-1 tumours showed a high density of PECAM-positive tube-like structures (indicated by arrows) compared to the HT1080/Scr tumours indicating a high level of angiogenesis (original magnification: 100X) **F.** The strong nuclear localization of HIF-1α seen in these tumours confirms a high level of *in situ* hypoxia (original magnification: 200X). **Hypoxia-priming enhances the** ***in vivo*** **tumour formation.** **G.** HT1080/Scr and HT/flNRP-1 cells – primed or not with hypoxia – were injected sub-cutaneously in the skin of NOD/SCID mice. The size of the tumours formed by the hypoxia-primed cells was significantly larger that their respective normoxic controls. Quantification of the tumour weights (N = 9) shows that hypoxia-priming significantly enhances the tumour formation by both HT1080/Scr as well as by the HT/flNRP-1 cells (Data are represented as mean± S.E.M., N = 9, ***p<.001 and **p<.01.) **H.** The kinetics of tumour formation by the hypoxia-primed cells at various time points was significantly higher compared to their normoxic counterparts (*p<0.05, **p<0.01, ***p<0.001).

From the various *in vitro* assays performed, it was evident that the hypoxia-mediated HIF-1α-dependent up-regulation of NRP-1 plays an active role in the angiogenic and tumorigenic properties of HT1080 cells. To validate our results *in vivo*, HT1080/Scr, HT/shNRP-1 and HT/flNRP-1 cells were injected subcutaneously in the flanks of the NOD/SCID mice and the tumour formation was monitored at regular intervals by measuring the tumour volume. In case of the HT/shNRP-1 cells, the tumour formation was completely abrogated (0/9 mice injected; Figure. 7A). The HT/flNRP-1 cells on the other hand formed the tumours very early (6^th^ day vs. 10^th^ day by the HT1080/Scr cells) and the tumour size was significantly larger compared to the HT1080/Scr cells (Figure. 7B; ***p<0.001). The measurement of tumour volume at various time points showed that the kinetics of the tumour formation by HT/flNRP-1 cells was significantly higher than that of the HT1080/Scr cells (Figure. 7C; ** p<0.01, *** p<0.001).

IHC experiments were performed on the paraffin sections of the HT/flNRP-1 tumours revealed that these tumours expressed various angiogenic markers viz. PECAM, VE-Cadherin, VEGF, NRP-1, VEFGF_165_ and VEGFR-2 at a very high level (Figure 7D). Immunostaining of the tumour sections with an antibody to PECAM revealed a higher micro-vessel density in the tumours formed by the HT/flNRP-1 cells than the HT1080/Scr cells (Figure. 7E). Consistent with the larger size of these tumours, the nuclear signal of HIF-1α was found to be much stronger in these tumour sections indicating a higher level of hypoxia (Figure 7F). The data suggested that NRP-1 enhances the angiogenic program in the HT1080 cells *in vivo* as well.

The difference in the behaviour of the HT1080/Scr, HT/shNRP-1 and HT/flNRP-1 cells with respect to their angiogenic and tumorigenic properties made it imperative to examine whether a difference in their growth kinetics and/or level of apoptosis in culture, especially under hypoxic conditions, could explain this differential behaviour. When assessed for these parameters, it was observed that these cells neither deferred in their growth kinetics (Figure. S2C) nor did they show any significant level of apoptosis in culture (Figure. S2D) under hypoxia, showing that the behavioural difference in these cells could principally be attributed to the difference in the level of NRP-1 in them, underscoring the importance of NRP-1 in these processes.

We further evaluated the expression of VEGFR-1, VEGFR-2 and NRP-2 in these cells by performing quantitative PCR experiments. Data showed that VEGFR-2 mRNA was significantly down-regulated in the HT/shNRP-1 cells while it was significantly up-regulated in the HT/flNRP-1 cells (Figure. S3A; 0.4 folds and 3.7 folds respectively; *** p<0.001) compared to the HT1080/Scr cells. The VEGFR-1 mRNA levels were found to be comparable in all of them (Figure. S3B), suggesting that perhaps its expression was not modulated by NRP-1. Surprisingly, NRP-2 mRNA was found to be up-regulated in HT/flNRP-1 cells (8 fold; Figure. S3C), but this up-regulation could not be solely attributed to the exogenous expression of NRP-1, as NRP-2 was found to be up-regulated (3 folds, Figure. S3C) in the HT/shNRP-1 tumor cells as well. These results suggest that NRP-1-VEGFR-2 axis played a major role in the VM of HT1080 cells.

### Hypoxia-priming Enhances Tumour Formation by HT1080 Cells

In the previously described experiments we had observed an enhanced tubulogenesis by the hypoxia-primed HT1080 cells. Here we examined whether hypoxia-priming offers an added advantage to the tumour cells in terms of an early tumour formation and a higher tumour growth *in vivo*. HT1080/Scr, HT/shNRP-1 and HT/flNRP-1 cells, grown in normoxia or hypoxia for 48 hours, were injected in the flanks of NOD/SCID mice. The hypoxia-primed HT1080/Scr cells gained an advantage over their normoxic counterparts in terms of the early onset (8^th^ day vs. 10^th^ day), the kinetics of tumour formation (Figure. 7H) as well as with respect to the tumour size (Figure. 7G; N = 9, **p<0.01). The HT/shNRP-1 cells failed to form tumours even after hypoxia-priming, once again underscoring the crucial role of NRP-1 in the tumour formation by the HT1080 cells. Consistent with the results obtained in the earlier experiments, an addition of chetomin in the incubation medium during hypoxia priming abrogated the tumour formation (Figure. S3D), suggesting that the hypoxia-mediated enhancement in the tumour formation was critically dependent on the HIF-1α-mediated transcription.

In the earlier experiment, we have observed that the normoxic HT/flNRP-1 cells formed the tumours earlier than the wild type cells and the tumour size was also larger. Interestingly, the tumours formed by the hypoxia-primed HT/flNRP-1 cells were even larger than their normoxic counterpart (Figure. 7G; N = 9; ***p<0.001) probably reflecting contributions from other hypoxia-induced responses.

### Hypoxia Modulates the Expression of Cancer Stem Cell Markers in the HT1080 Cells

**Figure 8:**
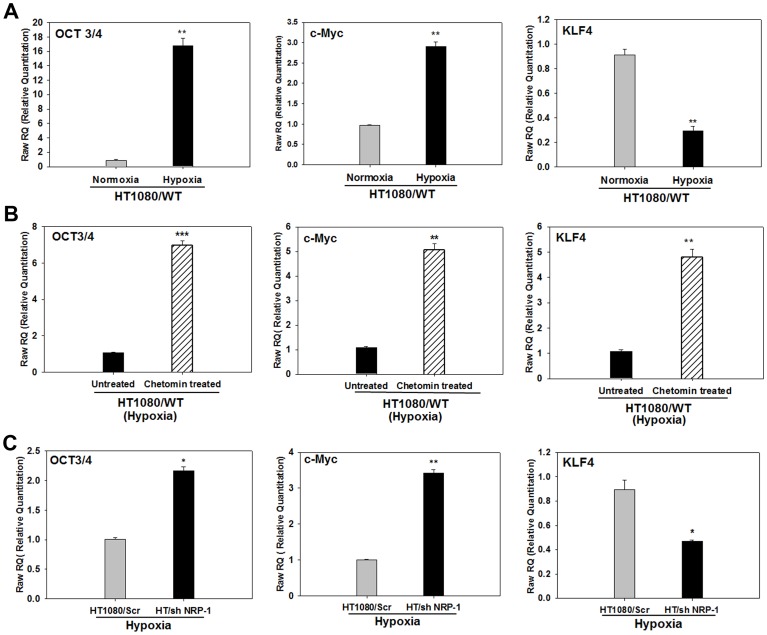
Hypoxia modulates the expression of cancer stem cell markers in the HT1080 cells. A. Quantitative PCR analyses for OCT3/4, c-Myc and KLF4 mRNA shows that hypoxia up-regulated the expression of OCT3/4 and c-Myc while down-regulates that of KLF4. **B.** Presence of chetomin in the incubation medium of cells incubated under hypoxia resulted in a further up-regulation of the expression of OCT3/4 and c-Myc mRNA indicting that these genes are independent of HIF-1α-mediated transcription and is under the control of a mechanism that is negatively regulated by the HIF-1α-mediated transcription. The down-regulation of KLF4 by hypoxia was however, rescued by chetomin showing that it was a HIF1-α-dependent process. **C.** Quantification of OCT3/4, c-Myc and KLF4 mRNA in the hypoxia-primed HT1080/Scr and HT/shNRP-1 cells shows that the expression of OCT3/4 and c-Myc is significantly higher in the hypoxia-primed HT/shNRP-1 cells compared to the hypoxia-primed HT1080/Scr cells, showing that the up-regulation of these genes by hypoxia is not only NRP-1 independent, but also gets further enhanced when NRP-1 is silenced.The suppression of KLF4 expression by hypoxia was partially rescued by silencing of NRP-1 indicating the role of HIF-1α-NRP-1 axis in its down-regulation by hypoxia. (Data in all panels are represented as mean ± S.E.M; N = 3 and *** p<0.001, ** p<0.01 and *p<0.5).

Presence of cancer stem cells has been linked to tumour progression. The HT1080 cells have been shown to form sarcospheres that express stem cell-related genes like Nanog, OCT3/4 and SOX2 [41]. Since hypoxia is known to promote a stem cell phenotype [42], we examined whether hypoxia regulated the expression of stem cell-associated genes in the HT1080 cells by performing quantitative PCR experiments. We found that the normoxic HT1080 cells expressed very low transcript levels for OCT3/4 and c-Myc, but incubation in hypoxic conditions increased their expression by ∼16 and ∼3 fold respectively (Figure. 8A). Conversely, the normoxic expression level of KLF4 was down-regulated ∼3 fold under hypoxic conditions (Figure. 8A). Nanog and SOX2 were not detected under either condition (data not shown). Incorporation of chetomin in the medium during hypoxia further up-regulated the expression of OCT3/4 (∼7 folds) as well as c-Myc (5 folds), indicating that their expression may be actively suppressed by HIF1α-mediated transcription in response to hypoxia (Figure. 8B). Suppression of KLF4 expression by hypoxia was, however, prevented by chetomin (∼4.5 folds increase), indicating that hypoxia down-regulates KLF4 via HIF-1α-dependent manner in these cells (Figure. 8B).

Since both NRP-1 and KLF4 were found to be regulated by hypoxia in a HIF-1α-dependent manner, we quantified the level of KLF4 in the HT/shNRP-1 cells under hypoxic conditions. We found that shRNA-mediated silencing of NRP-1 could not rescue the down-regulation of KLF4 by hypoxia (1.8 folds), indicating that NRP-1 was not involved in this process (Figure. 8C, right hand panel).

On the other hand, hypoxia treatment up-regulated the expression of OCT3/4 and c-Myc genes in the HT/shNRP-1 cells to significantly higher levels compared to the HT1080/Scr cells (Figure. 8C), indicating that regulation of these two stem cell-related genes was an NRP-1-independent event. The data suggest that in addition to the up-regulation of NRP-1 that in turn up-regulated the expression of the angiogenic molecules such as VEGF_165_, VEGFR-2 and PECAM, an up-regulation of OCT3/4 and c-Myc together with a down-regulation of KLF4 may also contribute towards the aggressive growth and angiogenesis of the HT1080 tumour under hypoxia.

However, the fact that the shRNA-mediated silencing of NRP-1 resulted in a complete abrogation of the *in vitro* as well as *in vivo* tumorigenesis and angiogenesis clearly underscores that NRP-1 plays a deterministic role in the vasculogenic mimicry and tumour growth of HT1080 cells.

## Discussion

Since the importance of angiogenesis in the tumour growth, survival and response to the therapy was realised [2], there has been a constant quest to identify the appropriate molecule/receptor that can be therapeutically targeted so as to suppress the tumour growth. Solid tumours invariably develop hypoxic regions as a consequence of their growth. A large number of genes involved in different steps and individual phenotypic processes involved in angiogenesis are known to be regulated by hypoxia [43]. A key molecule that plays an important role in this process is the hypoxia-inducible factor 1 (HIF-1α). The HIF-1α-mediated transcription plays a critical role in the tumour angiogenesis and growth, though some HIF-1α- independent mechanisms are also known to play an important role in the process. Chemical activation of HIF-1α by using prolyl hydroxylase inhibitor [44] was sufficient to induce angiogenic responses in a set of cell lines, including HT1080, under normoxia, clearly supporting its role in the tumour angiogenesis. Owing to its important role in the tumour angiogenesis, HIF-1α has been considered as a potential target to control tumour angiogenesis in multiple models [45].

HT1080, a human fibrosarcoma-derived cell line, is known to form aggressive tumours in immuno-compromised mice. It is frequently used as a model system for tumour angiogenesis. Surprisingly, the angiogenic mechanism utilised by these cells has not been investigated at the molecular level. A chemotactic attraction of the endothelial cells in response to the secretion of VEGF has been implicated in the angiogenic behaviour of HT1080 cells [46]. Accordingly, VEGF has been considered as an appropriate therapeutic target to curtail the tumour growth [47], [30]. Recently, the role of angiogenin in the *in vitro* tubule formation by HT 1080 cells has been demonstrated [48]. Our histo-pathological as well as immuno-histochemical staining clearly showed the presence of circles as well as ellipses (Figure. 1A) harbouring RBCs. As suggested by McDonald et al [49] in their illustration (Figure. 1B in the reference) these structures would only appear when a 3-dimentional anastomosing network of tubes or sinusoids gets cut at various planes of the section. Thus, the presence of such structures harbouring RBCs in the tumour sections confirms the vascular mimicry of these cells.

HT1080 cells have been shown to form cord-like structures on matrigel – a typical feature exhibited by vascular cells suggesting that perhaps these tumours use vasculogenic mimicry as the survival strategy [50]. We found that hypoxia-primed HT1080 as well as MDA-MB-231 cells formed robust tubules on matrigel. Similar results have also been reported by van der Schaft et al [7] with Ewing sarcoma cell lines, suggesting that the phenomenon of enhanced tubule formation in response to hypoxia may be exhibited by many tumour cell types. Present endeavour was undertaken to identify the mechanistic aspects of HT1080 tumor angiogenesis. We specifically investigated the hypoxia-mediated molecular events involved in the angiogenic behaviour of HT1080 cells. In order to demonstrate the vasculogenic mimicry of the HT1080 cells *in vivo*, we used HT1080 cells expressing a reporter gene, GFP, to clearly demarcate the tumour cells vis-à-vis host-derived endothelial cells. Expression of angiogenic markers by the GFP^+^ tumor cells, formation of blood-conducting vessels by the GFP-PECAM double positive cells and absence of PECAM^+^GFP^−^ cells in the tumor mass unambiguously demonstrated that indeed the process of vasculogenic mimicry was operative in the HT1080 tumours. We have clearly documented that the hypoxia-mediated HIF-1α-dependent up-regulation of NRP-1 not only confers the angiogenic properties on the HT1080 cells leading to vasculogenic mimicry, but also augments their tumour-forming ability, thereby acting as a double-edged sword in these cells. However, it may be interesting to see whether similar results can be obtained with other tumour-derived cell lines.

NRP-1 has been identified as a high affinity receptor for VEGF. Its role in the tumour growth and angiogenesis has been documented in several systems [51]. An exogenous expression of NRP-1 has been shown to induce a higher degree of angiogenesis in human dermal micro vascular endothelial cells and human umbilical vein endothelial cells [52], as well as in prostrate cell lines leading to the formation of larger tumours [53]. Our results are in complete agreement with these reports.

Neuropilin research has intensified recently. A link between NRP-1 expression, NF-κB activation, and mammosphere formation in breast cancer cells has been established [54]. Similar results have also been shown in human glioma stem-like cells [55] and skin tumors [56]. These findings suggest that NRP-1 has a key role in Cancer Stem Cell (CSC) formation and might be a good target for anti-CSC therapy. A recent study has identified differential NRP-1 expression on the surface of natural and induced Treg cells that suppress an effective anti-tumor immune response within tumor tissues [57]. NRP-1 expressed on these cells regulates the immunological anti-tumor control by guiding them into the tumor in response to tumor-derived VEGF. Accordingly, T cell-specific ablation of NRP-1 resulted in compromised melanoma growth [58]. NRP-1 was shown to orchestrate communications between myofibroblasts and soluble fibronectin that promote α5β1 integrin–dependent fibronectin fibril assembly, matrix stiffness, and tumor growth, underscoring its role in the activation of tumour microenvironment [59]. NRP-1 expression has been shown to control viability and proliferation of various cancer cells via interaction of its extra-cellular domain with the Epidermal Growth Factor receptor thereby promoting the ligand-induced signaling cascade that could be counteracted by the use of NRP-1-blocking antibodies and NRP-1 silencing [60]. Consistent with these reports, our data clearly showed that NRP-1 plays a deterministic role in the tumour growth and angiogenesis. Therapeutic targeting of a gene to inhibit tumour angiogenesis, and consequently, the tumour growth, usually involves silencing of the genes by siRNA or the use of specific neutralizing antibodies to angiogenic molecules like VEGF, angiopoietin etc. NRP-1 expression by the tumour cells has been known to promote tumour angiogenesis and progression [61], [53] and an exogenous expression of NRP-1 has been shown to be sufficient to drive angiogenesis [53]. NRP-1 along with its other family members like NRP-2 is, therefore, considered as a new target for cancer therapy [62], [63].

Hypoxia has been shown to up-regulate the expression of NRP-1 in certain normal cell types like the monkey choroid-retinal endothelial cells [64], ES cells [39] and also in neuroblastoma cells [65]. In the present study we have demonstrated that in HT1080 cells, hypoxia up-regulates the expression of NRP-1 in a HIF-1α-dependent manner and, more importantly, this up-regulated NRP-1 plays a deterministic role in their vasculogenic mimicry and tumour growth. Silencing of NRP-1 in the HT1080 cells completely abrogated the tumour growth, even after their priming with hypoxia, indicating that this is an excellent therapeutic target for containment of such tumours. It may be, however, necessary to examine this aspect using various tumour cells lines, including those that do not express NRP-1, so that broader conclusions can be drawn.

Several approaches have already been used to inhibit the NRP-1 function and, consequently block the pathological angiogenesis and tumour growth. Among these are antagonistic soluble NRP-1 [66], VEGF_165_-derived blocking peptides [67], [68], siRNA against NRP-1 [67], antibodies to NRP-1 [69] and recently developed synthetic small molecule inhibitors [70]. Pan *et al* showed that a combined blocking of NRP-1 and VEGF gives an additive effect to control tumour angiogenesis by interfering with the endothelial cell migration [71]. In our experiments we observed that the tumour cells themselves had acquired vascular cell-like properties *in vivo* as a consequence of HIF1-α-mediated up-regulation of NRP-1. NRP-1 not only conferred a vascular-like phenotype on the HT1080 cells, but also augmented their tumorigenic properties like matrigel invasion and anchorage-independent growth. The shRNA-mediated silencing of NRP-1 was found to be completely effective in abrogating the tumour growth. Up-regulated NRP-1 has been shown to be associated with carcinomas of various tissues including lung, breast, prostrate etc. [51]. Our data show that sarcomas may also show an up-regulated NRP-1 as a consequence of tumour hypoxia. It may be a worthwhile exercise to examine the expression of NRP-1 in all types of solid tumours and to target it therapeutically, if found to be up-regulated.

NRP-1 and NRP-2 are expressed in several types of tumour cells; in many cancers the expression of one or both has been correlated with tumour progression and/or poor prognosis [53], [72], [73]. In contrast to the endothelial cell system, where hypoxia has been reported to suppress the NRP-2 expression in a HIF-1α-dependent manner, in the HT1080 cells its expression was not affected by hypoxia, suggesting that NRP-2 may not have a significant role to play in the hypoxia-mediated up-regulation of angiogenic and tumorigenic behaviour of the HT1080 cells [74].

Mathieu *et al* have shown that hypoxia, through hypoxia-inducible factor (HIF), can induce an hESC-like transcriptional program, including the induced pluripotent stem cell (iPSC) inducers, OCT3/4, Nanog, SOX2, KLF4, c-Myc, and microRNA-302 in several cancer cell lines derived from multiple tissues [18]. In our study, we found that the HT1080 cells showed a high expression of KLF4 under normoxia that got down-regulated in the presence of hypoxia in a HIF-1α-dependent, but in an NRP-1-independent manner. Since its down-regulation by the hypoxia coincided with the up-regulation of vascular-like cell fate by the HT1080 cells, it is likely that under normoxia KLF4 acts as a negative regulator of angiogenesis. KLF2 and KLF4 are closely related KLF family members. KLF2 has been shown to be a potent inhibitor of angiogenesis [75], but such information about KLF4 has not been reported. Whether the down-regulation of KLF4 by hypoxia has a direct role in the promotion of tumor angiogenesis needs further investigation. Exposure of colorectal cancer cell lines to hypoxia has been shown to result in enhanced expression of miR-103/107 that targeted the metastasis suppressors KLF-4 and death-associated protein kinase (DAPK), conferring an invasive phenotype on them [76]. It will be interesting to examine whether such miR-mediated mechanism is operative in hypoxia-exposed HT1080 cells.

c-Myc is known to function as a master regulator angiogenesis in developmental processes as well as in tumours [19]. HIF-1α is known to counter the effects of normal c-Myc and effect its homeostatic regulation. On the other hand, dys-regulated c-Myc expressed in cancer cells cooperates with HIF-1α to produce VEGF that is an important mediator of tumor angiogenesis [20], [77]. Up-regulation of c-Myc by hypoxia and increased secretion of VEGF seen in our study suggest that HT1080 cells express dis-regulated c-Myc. The hypoxia-induced up-regulation of c-Myc and OCT3/4 in these cells was found to be an HIF-1α-independent event, indicating that such HIF1α-independent mechanisms may also contribute in the tumor angiogenesis. In the HT/shNRP-1 cells, the expression level of OCT3/4 and c-Myc was further augmented by hypoxia, suggesting that not only these genes are NRP-1-independent but also compete with NRP-1 for some factors involved in their transcriptional regulation. Nonetheless, silencing of NRP-1 alone resulted in a complete abrogation of tumour formation by the HT1080 cells, indicating that NRP-1 plays a dominant role in angiogenesis and HT1080 tumour growth.

In conclusion, our data show that the HT1080 cells respond to *in situ* hypoxia by adopting vasculogenic mimicry, in which the up-regulation of angiogenic program via HIF-1α-NRP-1 axis plays a critical role. The silencing of NRP-1 resulted in a complete abrogation of tumour formation, confirming its status as a promising therapeutic target for the control of tumour angiogenesis and growth.

## Materials and Methods

### Cell Culture

HT1080, MDA-MB-231 and MC3T3#24 cell lines were was purchased from ATCC (American Type Culture Collection, Manassas, VA, USA) and were maintained as per their instructions. The cells were incubated at 37°C in a humidified atmosphere containing 5% CO_2_. Hypoxia- treatment was given by placing the cells in a hypoxia chamber (Billups, Rothenberg Inc. Modular incubator chamber MIC-101 CA, USA) flushed with a gas mixture comprising 1% O_2_, 5% CO_2_ and 94% N_2_. The chamber was in turn placed in a humidified incubator (Thermo scientific; Asheville, NC, USA) set at 37°C.

In some experiments, chetomin (Sigma-Aldrich Louis, MO, USA) was added in the growth medium at a concentration of 100 nM during the incubation under hypoxia. Equivalent amount of DMSO (solvent) was added in the corresponding control cultures. Chetomin did not have any toxic effect on the cells.

### Growth Kinetics

The growth rate of the HT1080 cells was studied till 72 hours. 1×10^5^ cells were seeded in 35 mm dishes and the cultures were maintained under hypoxic (1% O_2_) or normoxic (20% O_2_) conditions. After every 24 hours, the cells from individual dishes (3 each) were harvested by trypsinization and a viable cell count was taken using the trypan blue dye exclusion method. A mean ± Standard Error of Means (S.E.M.) of triplicate cultures were determined.

### Western Blot Analysis

The western blot analyses were performed as described earlier [78]. Cells were lysed 48 hours after incubation under normoxic or hypoxic conditions. The protein samples (20 µg) were loaded on 8% polyacrylamide gel and the separated proteins were transferred to PVDF membrane overnight at 4°C. After blocking with 5% BSA in TBST buffer (8 mM Tris-HCL (pH 7.4), 135 mM NaCl and 0.05% Tween 20), the membranes were probed with primary antibodies [(NRP-1 and GFP antibodies (SCBT; CA, USA), β-actin antibody (Sigma) and HIF-1α antibody (Upstate, Milipore; MA, USA) were used at 1∶500 dilution (**Table S1**)] followed by appropriate horse radish peroxidase-conjugated secondary antibodies. The signals were detected using Lumiglo reagent (Cell Signaling; Danvers, MA, USA) and captured on Konica X-ray films. Densitometric analysis of the bands was done using Image J software (NIH) and the values were normalised with those obtained with beta-actin.

### Matrigel Invasion Assay

The *in vitro* matrigel invasion assay was performed using Bio-Coat Matrigel invasion assay system (BD Biosciences; San Jose, CA, USA), according to the manufacturer’s instructions. 1×10^4^ cells were suspended in their respective complete growth medium and seeded into the upper chambers of the Matrigel transwell inserts having a polycarbonate membrane base with 8-µm pores. After incubation for 18–20 hours, the upper surfaces of the membranes were gently wiped with cotton swabs to remove the non-invaded cells and the membranes were fixed and stained with 0.1% crystal violet solution to visualise the invaded cells. The number of invaded cells/field were counted under a phase contrast microscope (Olympus) using 10X objective and eye piece. Mean ± S.EM. of the cell number obtained in fifteen randomly selected, non-overlapping fields/well was determined for statistical analysis.

### Soft Agar Colony-formation Assay

Anchorage- independent growth ability of the cells was determined by assaying colony formation in soft agar as described below. Briefly, 1×10^4^ cells were suspended in a complete growth medium containing 0.3% agarose. The suspension was over layered on a pre-formed solidified layer of 0.6% agarose in the complete growth medium. The cells were fed every 2–3 days with 200 µl of complete growth medium. The colonies were counted after 20 days, under a phase contrast microscope using 10X objective and eyepiece. A mean ± S.E.M. of at least ten non-overlapping fields/plate was calculated for statistical analysis. The colony size was measured by performing image analyses using Image J software (NIH). Size of 50 colonies per plate was measured and the data were plotted as Mean ± S.EM.

### Immunofluorescence Staining

1×10^4^ cells were seeded on coverslips and were cultured under normoxic and hypoxic conditions for 48 hours. Cells were fixed with freshly prepared buffered 4% paraformaldehyde for 15 minutes, followed by a brief permeabilization with 0.1% Triton X-100, if required. After three washes with 1X PBS (pH 7.4), the cells were incubated with 1% BSA in PBS for 1 hour at room temperature (RT) to block nonspecific binding of the antibodies. Subsequently, the cells were incubated with specific primary antibody in 0.1% BSA for 1 hour at RT (NRP-1-1∶50 dilution (SCBT), HIF-1α 1∶100 dilution and VEGF_165_ (1∶100 dilution, Upstate, Millipore (**Table S1**), followed by incubation with appropriate, fluorescently-tagged secondary antibodies for 1 hour at RT in the dark. Cells were stained with DAPI (0.5 µg/ml; Invitrogen, Carlsbad, CA, USA) to demarcate the nuclei. Cells were washed thrice with 1X PBS before mounting in mounting media supplemented with an anti-fade. Imaging was done on confocal laser-scanning microscope (Carl Zeiss, Jena, Germany, LSM 510 META; 63X/oil/1.4NA objective) using AIM 4.2 software or on Leica microscope (Leica SP5II, Leica microsystem, Germany, LAS AF, 63X/oil/1.4A objective). Image analysis was done using LSM-5 or LAS AF Image examiner.

### Matrigel Tubeformation Assay

The assay was performed using growth factor-reduced Matrigel from BD [79]. Matrigel was thawed overnight at 4°C and was diluted (1∶1) with phenol red-free DMEM without serum. 200 µl of the diluted matrigel was used to coat the wells of a 24 well plate and was allowed to polymerise for 2 hours at 37°C. After equilibrating the gel with the complete growth medium, 2×10^4^ cells were seeded in each well and incubated for 20 hours. The tube formation was monitored under a phase contrast microscope (Olympus) and imaged at various time points using Olympus camedia 5060 camera. Tubule length at various time points was measured by performing image analyses using Image J software (NIH). Length of 40 tubules/plate was measured and the data were plotted as Mean ± S.EM.

### Detection of Apoptosis by Annexin V Staining

1×10^5^ cells of HT1080/WT/GFP, HT/fl NRP-1 and HT/sh NRP-1 were seeded in 35 mm dishes and were incubated under hypoxic conditions for 48 and 72 hrs. After incubation, the cells were collected by trypsinization, washed once with chilled 1X PBS and resuspended in 500 µl of 1X binding buffer (10 mM HEPES, 140 mM NaCl and 2.5 mM CaCl_2_). 10^5^ cells were used for the staining. Since HT1080/WT/GFP and HT/flNRP-1 cells were GFP positive, they were incubated with Annexin V-PE for 15 minutes at RT in dark, whereas the HT/shNRP-1 clone express RFP, therefore they were incubated with Annexin V-FITC. After 15 minutes, 5 µl of 7-AAD dye (0.25 µg) was added just before acquisition of the cells on FACS Canto II. Data were analysed using FACS Diva software (BD).

### Xenograft Model

Protocols used in the animal experimentation have been approved by the institutional animal ethics committee [Institute’s Animal Ethics Committee (IAEC) of NCCS]. The NOD.CB17-Prkdcscid/J (NOD/SCID) mice (Jackson laboratories, Bar Harbor, USA ) were bred in the animal house, kept in micro-isolators and fed with sterile food and acidified water. For tumour formation assay 1×10^6^ cells (HT1080/Scr, HT/shNRP-1, HT/flNRP-1 and HT1080/WT/GFP) were suspended in 75 µl of complete medium, mixed with an equal volume of phenol red-free growth factor-reduced matrigel (BD), and injected subcutaneously in the flanks of mice (9 mice per group). The tumour volume was measured at different time points with the help of a Vernier Calliper. The Length and the width of the tumor were measured and the volume was calculated using the formula:
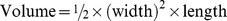



The mice were sacrificed on 20^th^ days after tumour cell implantation and the tumours formed were extracted and weighed after carefully removing the extraneous tissues. They were fixed in buffered formalin and processed for paraffin sectioning.

### Immunohistochemistry

Paraffin-embedded tissues were cut into 4-µm-thick sections. The sections were deparaffinized, hydrated in graded alcohol (100% to 30%) for 20 minutes each and rinsed with 1X PBS. Antigen-retrieval was done by heating the sections in 10 mM citrate buffer (pH 6.0) for 10 minutes in a microwave oven. The endogenous peroxidase activity was blocked by incubating the sections in 0.3% hydrogen peroxide prepared in methanol for 30 minutes at RT in dark. Non-specific binding was blocked by 2.5% Horse serum (Vector Laboratories, Burlingame, CA, USA) for 1hour at RT. After blocking, the sections were incubated with specific antibodies (**Table S2**) overnight at 4°C in a moist chamber. Next day the sections were washed and incubated with biotin-conjugated goat/rabbit/mouse-secondary antibody (Vector Lab) for 30 minutes at RT. After 2 washes with 1X PBS, HRP- conjugated streptavidin (Vector lab) was added and the slides were incubated for 15 minutes at RT. Slides were then developed using VECTOR VIP (purple) from Vector Laboratories for 10 minutes. The tumour sections were always made in duplicate on each slide and one section was used as a negative control (NC), wherein the primary antibody was not added, while the other was used as an experimental one. All the slides were counterstained with haematoxylin for 5 minutes, dried and mounted using permanent mountant (Vector Lab). For double IHC experiments, the sections were sequentially stained with an anti-GFP antibody, a biotin-labelled secondary antibody and finally with HRP-conjugated streptavidin. The GFP signal was detected by using DAB substrate (Brown). These sections were then re-processed for IHC with an anti-PECAM antibody. The PECAM signal was detected using VECTOR VIP substrate (purple). Additional controls without adding one of the primary antibodies (either anti-GFP or anti-PECAM) were also kept to ensure specificity of the signal.

Images were captured using Nikon microscope (Nikon Eclispse 80*i*, Germany) equipped with a camera and images obtained were analysed using Image Pro plus software.

### Silencing of NRP-1 Using shRNA-mediated Transfection

A set of four unique shRNA constructs directed against various regions of human NRP-1 mRNA were purchased from Origene (HuSH 29mer shRNA constructs against human NRP-1, Origene technologies, Inc.; Rockville, MD, USA). Cells were transfected with a mixture of all four shRNA plasmid constructs (1 µg each). HT1080 cells transfected with the scrambled negative control non-effective shRNA cassette-containing expression vector were used as the experimental control (HT1080/Scr). The transfections were done using lipofectamine 2000 reagent (Invitrogen) on 60–70% confluent cells cultured in a six well plate according to the manufacturer’s instructions. After transfection, 2 µg/mL of puromycin (Sigma) was added to the medium to select the antibiotic-resistant clones. The independent clones were picked up and further purified by sorting on FACS ARIA (BD) using yellow-green laser (excitation λ 561 nm and emission λ 582 nm) to gate the RFP positive population. The clones were characterised by real time PCR, western blotting and immunofluorescence experiments to assess the level of silencing of NRP-1.

### Generation of Full Length NRP-1 Clone in HT1080

NRP-1 was cloned in the eGFP-N1 vector (Clontech, CA, USA) using BglII and SalI restriction sites. Kozak consensus sequence was added to upstream of the ATG start codon.

The primer sequences used for cloning were as follows.

CL_NRP1/Bgl II F- 5′ATTAGATCTTCGCCACCATGGAGAGGGGGCTGCCGCT3′

CL_NRP1/Sal1 R-5′ATAGTCGACGCCTCCGAATAAGTACTCTGTGTATTCAG3′

Full length NRP1 (2.77 kb) gene was amplified from the cDNA of HT1080 cells. Purified PCR products and eGFP-N1 vector were digested with Bgl II and Sal I restriction enzyme (New England Biolabs; MA, USA). The digested vector was subjected to PEG/NaCl purification and the NRP-1 fragment was purified by phenol/chloroform precipitation. The NRP-1 fragment was ligated with the digested eGFP-N1 using T4 DNA ligase enzyme. The ligated plasmid was transformed into DH5α competent cells and the colonies were selected on LB agar plates containing kanamycin at 100 µg/ml concentration (Sigma). The positive clones were identified by performing colony PCR. The insertion of NRP-1 was confirmed using restriction digestion analysis, PCR experiments and the sequences were confirmed by automatic sequencer (3730 XL DNA analyser, ABI). Stable clones of HT1080 cells expressing full length NRP-1 or the control vector eGFP-N1, were generated by transfection as described earlier, except that the selection antibiotic was G418 (USB; Cleaveland, OH, USA at 600 µg/ml conc.). The independent clones were expanded and purified by sorting on FACS ARIA using blue laser (excitation λ 488 nm and emission λ 530 nm) to gate the GFP positive population.

### Gene Expression Studies

The mRNA was isolated from cells using mRNA isolation kit (Dynal/InVitrogen), and was reverse transcribed using Superscript II (Invitrogen). Real time PCR experiments were performed using inventoried Taqman gene-expression assays (Applied Biosystems; Foster City, CA,USA) or gene-specific primers using Syber Green (Invitrogen ) chemistry to quantify the gene expression on ABI 7500 Fast machine (Applied Biosystems). GAPDH was used as the normalizer for analysis (**Table S3**). An RT^neg^ set was kept to detect genomic contamination. The primers were designed using Primer-3 software V0.4.0 (http://prodo.wi.mit.edu/primer3/).

### Statistical Analysis

Statistical analysis was performed using one-way repeated-measure variance analysis (One-Way RM ANOVA) using Sigma Stat software (Jandel Scientific Corporation, San Raphael, CA). The values were represented as mean ± S.E.M. p value ≤0.05 was considered as statistically significant.

## Supporting Information

Figure S1 **A.** Quantification of tubule length at various time-points shows that the hypoxia-primed HT1080 cells form significantly longer tubules compared to those formed by the normoxic cells (N = 40, ***p<0.001). **B.** Hypoxia-primed MDA-MB-231 cells form robust tubules on matrigel in a chetomin-sensitive manner. Data indicate the involvement of HIF1-α-mediated trancritpion in the enhancement of tubule formation by these cells as well. (Original magnification:60X). **C.** An image of a stable clone of HT1080 cells transfected with eGFP-N1 plasmid is depicted. The GFP is seen both in cytoplasm and nuclei. The intensity of the GFP signal is high in the nuclear region. **D.** NRP-2 may not be involved in the hypoxia-mediated angiogenic response of the HT1080 cells. Expression of NRP-1 and NRP-2 mRNA was quantified by real time PCR experiments. Hypoxia significantly up-regulated the level of NRP-1, but had no effect on the level of NRP-2. (N = 3 **p<0.01). **E.** Quantification of tubule length performed by image analysis using by Image-J software showed that the hypoxia-primed HT1080/Scr cells formed significantly longer tubules compared to those formed by their normoxic counterparts. (N = 40, ** p<0.01) The HT/shNRP-1 cells did not form tubules even after hypoxia-priming indicating the critical role of NRP-1 in the process. **F.** The tubules formed by the HT/flNRP-1 were significantly longer compared to the HT1080/Scr cells at both 4 and 6 hour time points. After this point the HT/flNRP-1 cells aggressively invaded the matrigel. (N = 40, # p<0.05, ## §§ and ** p<0.01).
(TIF)

Figure S2 **A.** The quantification of the colony size performed by image analysis using Image J (NIH) software shows that the HT/flNRP-1 cells form significantly larger colonies compared to those formed by HT1080/Scr cells. The HT/shNRP-1 cells formed very small colonies. **B.** The graph illustrates the enhanced clonogenic properties of HT/flNRP-1 cells compared to HT1080/Scr and HT/shNRP-1 cells. (N = 3, * p<0.05, **p<0.01). **C.** Growth kinetics of HT1080/Scr, HT/flNRP-1 and HT/shNRP-1 cells under hypoxic conditions is illustrated. It is seen that all the three cell types exhibited similar growth kinetics under hypoxia. **D.** Level of apoptosis in HT1080/WT/GFP, HT/flNRP-1 and HT/shNRP-1 cells grown under hypoxic conditions for 48 and 72 hours was evaluated by Annexin V staining. The cells did not show any significant level of apoptosis at either time point and there was no difference between the three cell types analysed.
(TIF)

Figure S3 **Quantitative PCR experiments were performed on the cDNA prepared from the lysates of HT1080/Scr, HT/shNRP-1 and HT/flNRP-1 cells.** **A.** VEGFR-2 expression was significantly up-regulated in HT/flNRP-1 and down- regulated in HT/shNRP-1 cells compared to the HT1080/Scr cells. **B.** VEGFR-1 expression was not significantly different in these cells. **C.** NRP-2 expression was found to be significantly up-regulated in HT/flNRP-1 cells compared to HT1080/Scr cells, but this effect could not be attributed to NRP-1 as the HT/shNRP-1 cells also showed higher levels of NRP-2. **D.** The image shows the tumours formation in the NOD/SCID mice. Dotted circles indicate the site of injection. The tumour formation by the hypoxia-primed HT1080/Scr cells was abrogated (N = 9) when the cells were incubated under hypoxia in the presence of chetomin, showing that the tumour formation by the hypoxia-primed HT1080/Scr cells critically depends on the HIF-1α-mediated transcription. The HT/shNRP-1 cells did not form tumour even after priming with hypoxia (N = 9).
(TIF)

Table S1 **List of the antibodies used for Western blotting and Immunofluorescence experiments.**
(DOC)

Table S2 **List of antibodies used in Immunohistochemistry staining experiments.**
(DOC)

Table S3 **List of Primers used for quantitative PCR experiments.**
(DOC)
