# Supplementary figures and images for: Vasculogenic Mimicry of HT1080 Tumour Cells In Vivo: Critical Role of HIF-1α-Neuropilin-1 Axis
Source: PLoS One. 2012 Nov 21;7(11):e50153. doi: 10.1371/journal.pone.0050153 (PMC3504006; doi:10.1371/journal.pone.0050153)

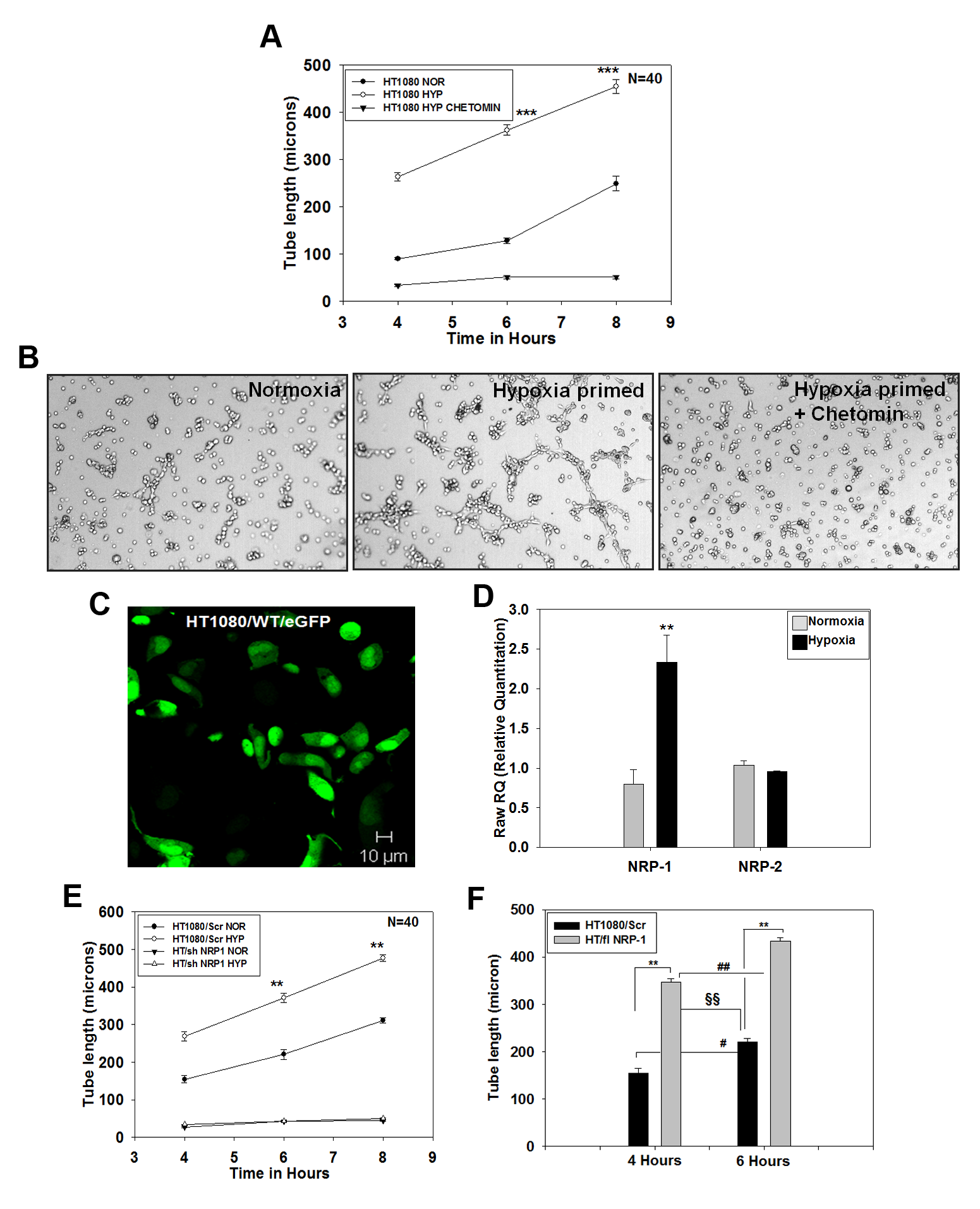

Supplement: Figure S1 — A. Quantification of tubule length at various time-points shows that the hypoxia-primed HT1080 cells form significantly longer tubules compared to those formed by the normoxic cells (N = 40, ***p<0.001). B. Hypoxia-primed MDA-MB-231 cells form robust tubules on matrigel in a chetomin-sensitive manner. Data indicate the involvement of HIF1-α-mediated trancritpion in the enhancement of tubule formation by these cells as well. (Original magnification:60X). C. An image of a stable clone of HT1080 cells transfected with eGFP-N1 plasmid is depicted. The GFP is seen both in cytoplasm and nuclei. The intensity of the GFP signal is high in the nuclear region. D. NRP-2 may not be involved in the hypoxia-mediated angiogenic response of the HT1080 cells. Expression of NRP-1 and NRP-2 mRNA was quantified by real time PCR experiments. Hypoxia significantly up-regulated the level of NRP-1, but had no effect on the level of NRP-2. (N = 3 **p<0.01). E. Quantification of tubule length performed by image analysis using by Image-J software showed that the hypoxia-primed HT1080/Scr cells formed significantly longer tubules compared to those formed by their normoxic counterparts. (N = 40, ** p<0.01) The HT/shNRP-1 cells did not form tubules even after hypoxia-priming indicating the critical role of NRP-1 in the process. F. The tubules formed by the HT/flNRP-1 were significantly longer compared to the HT1080/Scr cells at both 4 and 6 hour time points. After this point the HT/flNRP-1 cells aggressively invaded the matrigel. (N = 40, # p<0.05, ## §§ and ** p<0.01). (TIF) [file pone.0050153.s001.tif]

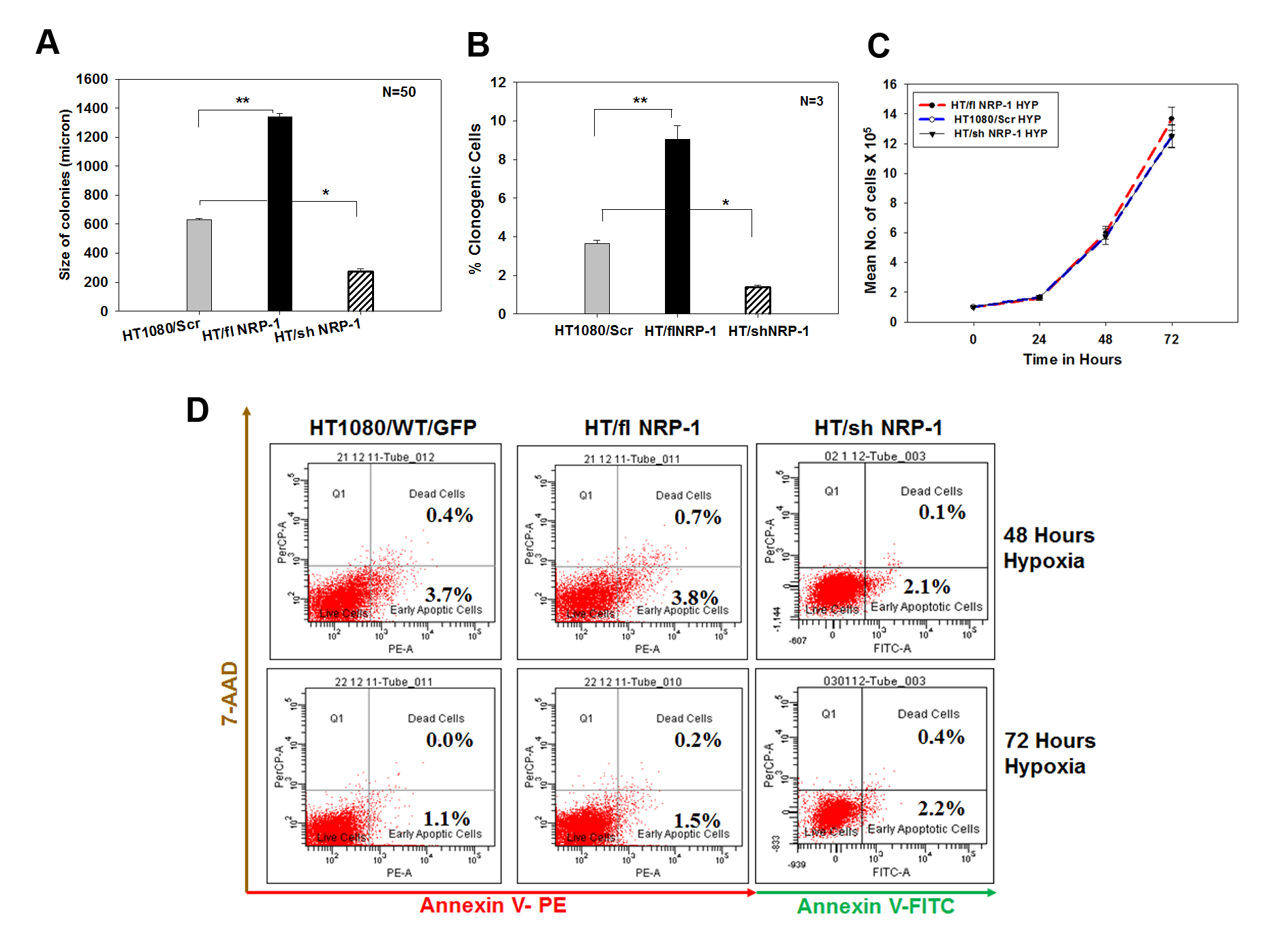

Supplement: Figure S2 — A. The quantification of the colony size performed by image analysis using Image J (NIH) software shows that the HT/flNRP-1 cells form significantly larger colonies compared to those formed by HT1080/Scr cells. The HT/shNRP-1 cells formed very small colonies. B. The graph illustrates the enhanced clonogenic properties of HT/flNRP-1 cells compared to HT1080/Scr and HT/shNRP-1 cells. (N = 3, * p<0.05, **p<0.01). C. Growth kinetics of HT1080/Scr, HT/flNRP-1 and HT/shNRP-1 cells under hypoxic conditions is illustrated. It is seen that all the three cell types exhibited similar growth kinetics under hypoxia. D. Level of apoptosis in HT1080/WT/GFP, HT/flNRP-1 and HT/shNRP-1 cells grown under hypoxic conditions for 48 and 72 hours was evaluated by Annexin V staining. The cells did not show any significant level of apoptosis at either time point and there was no difference between the three cell types analysed. (TIF) [file pone.0050153.s002.tif]

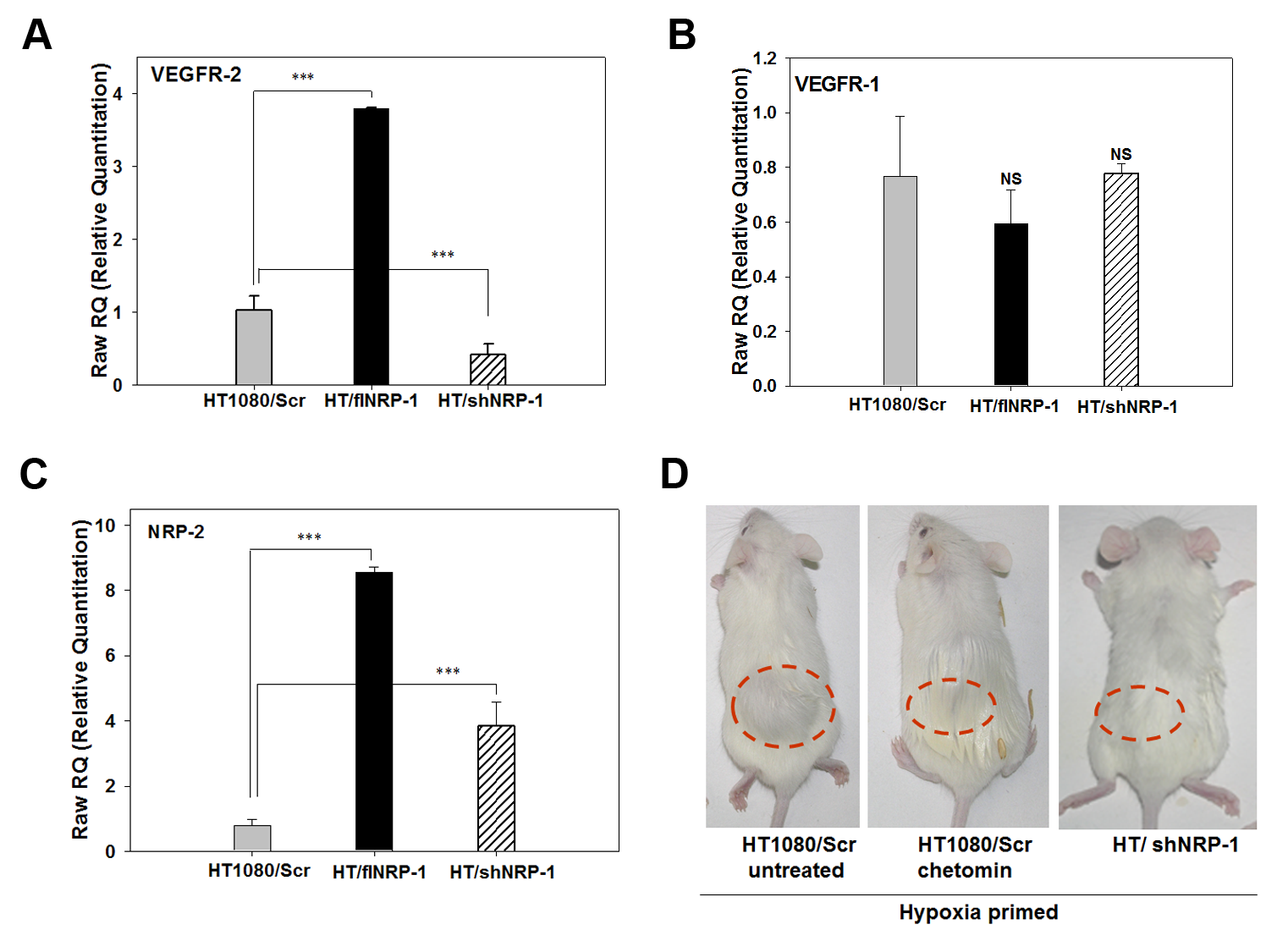

Supplement: Figure S3 — Quantitative PCR experiments were performed on the cDNA prepared from the lysates of HT1080/Scr, HT/shNRP-1 and HT/flNRP-1 cells. A. VEGFR-2 expression was significantly up-regulated in HT/flNRP-1 and down- regulated in HT/shNRP-1 cells compared to the HT1080/Scr cells. B. VEGFR-1 expression was not significantly different in these cells. C. NRP-2 expression was found to be significantly up-regulated in HT/flNRP-1 cells compared to HT1080/Scr cells, but this effect could not be attributed to NRP-1 as the HT/shNRP-1 cells also showed higher levels of NRP-2. D. The image shows the tumours formation in the NOD/SCID mice. Dotted circles indicate the site of injection. The tumour formation by the hypoxia-primed HT1080/Scr cells was abrogated (N = 9) when the cells were incubated under hypoxia in the presence of chetomin, showing that the tumour formation by the hypoxia-primed HT1080/Scr cells critically depends on the HIF-1α-mediated transcription. The HT/shNRP-1 cells did not form tumour even after priming with hypoxia (N = 9). (TIF) [file pone.0050153.s003.tif]
