# Supplementary material for: Vasculogenic Mimicry of HT1080 Tumour Cells In Vivo: Critical Role of HIF-1α-Neuropilin-1 Axis
Source: PLoS One. 2012 Nov 21;7(11):e50153. doi: 10.1371/journal.pone.0050153 (PMC3504006; doi:10.1371/journal.pone.0050153)
Supplement: Table S1 — List of the antibodies used for Western blotting and Immunofluorescence experiments. (DOC) [file pone.0050153.s004.doc]

**Table S1 -Antibodies used for Western blotting and Immnuofluorescence experiments.**

| *Antigen* | *Clone/Cat#* | *Distributor* |
| --- | --- | --- |
| HIF-1α | 07-1585 | Upstate, Millipore (MA,USA) |
| NRP-1 | C-19,sc-7239 | SCBT (CA, USA) |
| GFP | B-2, sc-9996 | SCBT |
| β-Actin | A5441 | Sigma (Louis,USA) |
| VEGF_165_ | 07-1419 | Upstate, Milipore |
