# Supplementary material for: Vasculogenic Mimicry of HT1080 Tumour Cells In Vivo: Critical Role of HIF-1α-Neuropilin-1 Axis
Source: PLoS One. 2012 Nov 21;7(11):e50153. doi: 10.1371/journal.pone.0050153 (PMC3504006; doi:10.1371/journal.pone.0050153)
Supplement: Table S2 — List of antibodies used in Immunohistochemistry staining experiments. (DOC) [file pone.0050153.s005.doc]

**Table S2- Antibodies used for Immunohistochemistry studies.**

| Antigen | Clone/Cat# | Distributor |
| --- | --- | --- |
| HIF-1α | H-206, sc-10790 | SCBT |
| PECAM | C-20,sc-1505 | SCBT |
| VEGF | A-20,sc-152 | SCBT |
| NRP-1 | C-19,sc-7239 | SCBT |
| VEGF_165_ | 07-1419 | Upstate, Milipore |
| VEGFR-2 | C-1158,sc-504 | SCBT |
| VE-Cadherin | A-8, Sc-9989 | SCBT |
