# Supplementary material for: Vasculogenic Mimicry of HT1080 Tumour Cells In Vivo: Critical Role of HIF-1α-Neuropilin-1 Axis
Source: PLoS One. 2012 Nov 21;7(11):e50153. doi: 10.1371/journal.pone.0050153 (PMC3504006; doi:10.1371/journal.pone.0050153)
Supplement: Table S3 — List of Primers used for quantitative PCR experiments. (DOC) [file pone.0050153.s006.doc]

**Table S3- List of Primers used for quantitative PCR studies.**

| **Name** | **Sequence 5' to 3'** | **Description and GenBank accession number** | **Product Size** |
| --- | --- | --- | --- |
| GAPDH F | ACTGCCACCCAGAAGACTGT | Glyceraldehyde-3-phosphate Dehydrogenase | 151 bp |
| GAPDH R | CCATGCCAGTGAGCTTCC | Homo sapiens [NM_002046.3] |  |
| VEGF165 F | GAAGTTTCGCAGACCTGACA | Secreted VEGF165 | 139 bp |
| VEGF165 R | ATTCAACTCCTCGCTTTCCA | Homo sapiens [NM_0010253682] |  |
| VEGFR-2 F | AGATGACAACCAGACGGACA | Vascular endothelial growth factor receptor-2 | 137 bp |
| VEGFR-2 R | AGCCTTCAGATGCCACAGAC | Homo sapiens [NM_002253.2] |  |
| VEGFR1 F | TCACCTGGACTGACAGCAAA | Vascular endothelial growth factor receptor-2 | 118 bp |
| VEGFR1 R | CAGCTGGAATGGCAGAAACT | Homo sapiens [NM_002019.4] |  |
| NRP-1 F | TGACACTGCCACCTCTGACT | Neuropilin-1 | 152 bp |
| NRP-1 R | ATGAAATGCTTGGGAACTGC | Homo sapiens [NM_003873.5] |  |
| NRP-2 F | CCAACGTCAAGCATCTCAAA | Neuropilin-2 | 189 bp |
| NRP-2 R | CTTTAGCTTCGGGTCAATGC | Homo sapiens [NM_003872.2] |  |
|  | **Taqman assays** |  |  |
| HIF-1α | Hs00936368_m1 |  |  |
| PECAM | Hs00169777_ml |  |  |
| KLF4 | Hs00358836_ml |  |  |
| OCT3/4 | Hs00742896_ml |  |  |
| Myc | Hs00153408_ml |  |  |
| GAPDH | Hs99999905_m1 |  |  |

**Quantitative PCR:**

Real time PCR was carried out on ABI 7500 Fast Sequence Detection System (Applied Biosystems) using inventoried Taqman gene expression assays (assay on demand) or gene-specific primers using SYBR green (InVitrogen) chemistry to quantify gene expression. Primers were designed using Primer-3 tool V0.4.0 (<http://prodo.wi.mit.edu/primer3/>) and a melting curve analysis was carried out to validate specific amplification of genes. Ct values were normalized with those obtained from the amplification of GAPDH.

PCR Conditions for DNA sequencing were as follows:

Denaturation: 96°C 10 secs

Annealing: 50°C 5 secs

Extension: 60°C 1 mins

25 Cycles were run.
